# A Systematic Review of Hardware-Accelerated Compression of Remotely Sensed Hyperspectral Images

**DOI:** 10.3390/s22010263

**Published:** 2021-12-30

**Authors:** Amal Altamimi, Belgacem Ben Youssef

**Affiliations:** 1 Department of Computer Engineering, King Saud University, P.O. Box 51178, Riyadh 11543, Saudi Arabia; 436204168@student.ksu.edu.sa; 2 National Satellite Technology Center, Space and Aeronautic Research Institute, King Abdulaziz City for Science and Technology, P.O. Box 8612, Riyadh 12354, Saudi Arabia

**Keywords:** hyperspectral image compression, hardware accelerators, remote sensing, power requirement, throughput, compression ratio, systematic review

## Abstract

Hyperspectral imaging is an indispensable technology for many remote sensing applications, yet expensive in terms of computing resources. It requires significant processing power and large storage due to the immense size of hyperspectral data, especially in the aftermath of the recent advancements in sensor technology. Issues pertaining to bandwidth limitation also arise when seeking to transfer such data from airborne satellites to ground stations for postprocessing. This is particularly crucial for small satellite applications where the platform is confined to limited power, weight, and storage capacity. The availability of onboard data compression would help alleviate the impact of these issues while preserving the information contained in the hyperspectral image. We present herein a systematic review of hardware-accelerated compression of hyperspectral images targeting remote sensing applications. We reviewed a total of 101 papers published from 2000 to 2021. We present a comparative performance analysis of the synthesized results with an emphasis on metrics like power requirement, throughput, and compression ratio. Furthermore, we rank the best algorithms based on efficiency and elaborate on the major factors impacting the performance of hardware-accelerated compression. We conclude by highlighting some of the research gaps in the literature and recommend potential areas of future research.

## 1. Introduction

Hyperspectral Imaging (HSI) is an enabling technology for a variety of remote sensing applications related to intelligence, commerce, agriculture, military, and even humanitarian purposes. Such applications include environmental monitoring [1], agricultural field observation [2], geological mapping [3], and mineral exploration [4], to name just a few. It has been steadily growing over the last few years. According to the research conducted by BCC (Business Communications Company, Wellesley, MA, USA), the growth of the global market for HSI is expected to increase at a Compound Annual Growth Rate (CAGR) of 14.7% for the period 2018–2023, from $104.0 million in 2018 to $206.2 million in 2023 [5].

**Figure 1:**
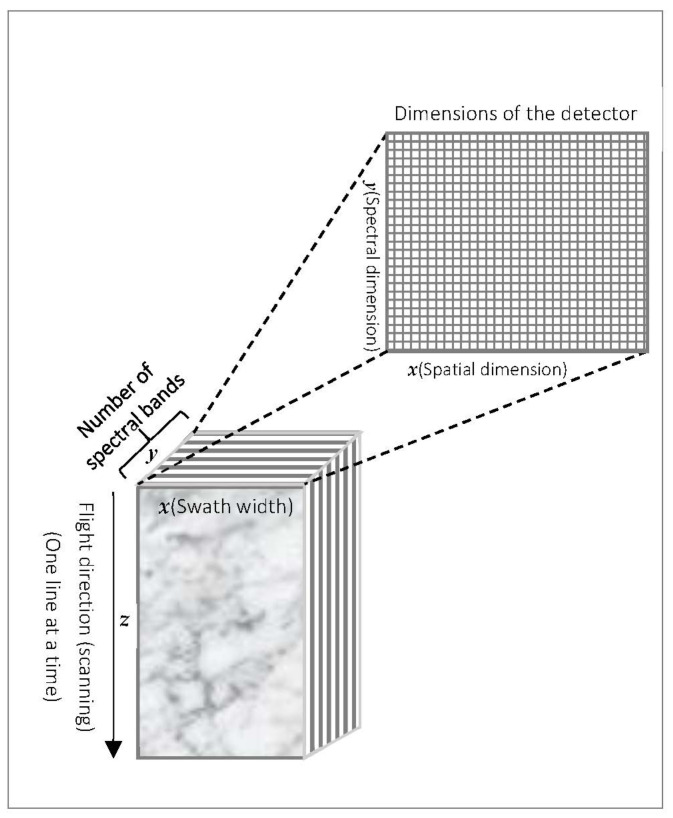
Conceptual view of the construction of hyperspectral images by a pushbroom scanner (not shown) during airborne flights.

The richness of information in hyperspectral images and the enhancements in sensor performance present an ever-increasing challenge due to the large size of hyperspectral data. For instance, the Airborne Visible InfraRed Imaging Spectrometer (AVIRIS) produces data as large as 16 Gigabytes per day [6]. The Atmospheric InfraRed Sounder (AIRS) is not far from the latter and can yield about 12 Gigabytes of data per day [6]. Typically, hyperspectral images consist of hundreds of contiguous bands, and the number of these bands depends on the detector resolution (see Figure 1). As illustrated in this figure, the *y* dimension represents the number of bands, and the *x* dimension corresponds to the swath width of the scene. The spatial dimensions (*x* and *z*) of the hyperspectral image are constructed one scan line at a time during flight time. According to [7], the number of bands of recognized hyperspectral imagers is as follows: (1) as many as 316 bands are acquired by the two payloads carried in the Indian Hyperspectral Imaging Satellite (HySIS); (2) 240 bands are collected by the Italian space agency’s satellite called no other than PRISMA, for PRecursore IperSpettrale della Missione Applicativa; (3) 220 bands are collected by the Hyperion imager onboard NASA’s Earth Observation satellite (EO-1); and (4) 232 bands are acquired by the German mission, known as the Environmental Mapping and Analysis Program (EnMAP). These significant band acquisitions result in large three-dimensional hyperspectral images, which make their onboard compression mandatory, especially for small satellites where the platforms are confined to limited storage capacity, weight, and power budget.

The adoption of small satellites began in the year 2000 with the emerging era of “new space” as Stanford University launched its microsatellite, called the Orbiting Picosat Automated Launcher (OPAL), containing six picosatellites [8]. Picosatellites such as CubeSats are excellent platforms for education as well as technology demonstration and are thus extremely valuable for countries without fully funded space programs. They offer benefits over larger satellites in terms of cost, development time, and payload modularity [9]. Small satellites also require smaller and lower-cost launch vehicles. Their onboard computers generally perform other tasks in addition to data acquisition and manipulation. These tasks include attitude determination and control, telecommand execution or dispatching, onboard time synchronization and distribution, and failure detection. Such tasks need to be prominently considered when designing the onboard computers for performance and power [10]. The latter two factors constitute the key drivers for the ever-increasing number of research studies on efficient compression utilizing parallel processing architectures suitable for onboard installation such as Graphics Processing Units (GPUs) and Field-Programmable Gate Arrays (FPGAs). GPUs and FPGAs have attracted much attention in High-Performance Computing (HPC) research seeing that the single-precision floating-point performance has reached more than ten Tera Floating-Point Operations Per Second (TFLOPS), far exceeding the computational performance of a Central Processing Unit (CPU) [11]. Digital Signal Processors (DSPs) also have a higher performance to cost ratio when compared to CPUs while requiring less power [12]. However, their level of performance is still far from what we can obtain using FPGAs and GPUs. The latter two hardware platforms are commonly used in the acceleration of image and signal processing applications, HPC-based simulations, and machine learning models. For instance, Microsoft has been using FPGAs to speed up search engines and deep learning models for cloud services [13]. Amazon’s cloud service provides HPC platforms in which GPUs and FPGAs are utilized as accelerators [11]. Google has also been using GPUs to speed-up machine learning for their systems [11].

In this paper, we present a systematic review of hardware-accelerated compression algorithms of remotely sensed hyperspectral images over a period spanning slightly over two decades. By hardware-accelerated, we mean the use of hardware accelerators such as FPGAs, GPUs, System-On-Chips (SoCs), and DSPs in the implementation and speeding up of compression algorithms for HSI. The main objectives of this work are to use the results of the review to answer the following four research questions (RQs):

- RQ1: What are the main hardware platforms and HSI datasets used to accelerate and evaluate HSI compression algorithms in remote sensing applications?
- RQ2: What are the different HSI compression algorithms and their classes that are accelerated in hardware?
- RQ3: What are the comparative performance results, obtained thus far, of the hardware-accelerated HSI compression algorithms?
- RQ4: What are some of the other pertinent factors that can impact the onboard implementation and utilization of hardware-accelerated HSI compression algorithms?

As a result of addressing these research questions, the contributions of this paper are as follows:

1. To describe the available hardware-accelerated compression algorithms of remotely sensed hyperspectral images, their implementation platforms, and their datasets;
2. To provide a comparative analysis of the collected studies against multiple metrics such as throughput, power requirement, compression ratio, and efficiency;
3. To discuss the major factors impacting the efficient development and continued progress in this important area;
4. To identify the related research gaps and present recommendations for future research work.

**Figure 2:**
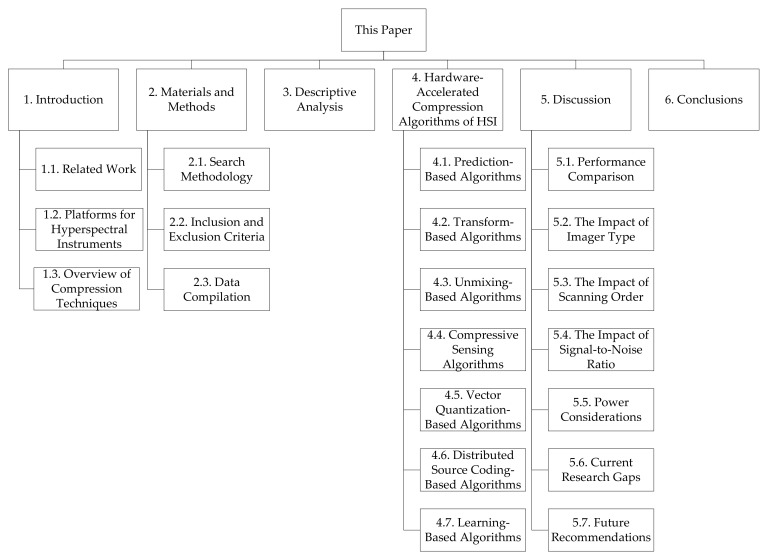
Organization of this article as a tree structure to help readers navigate through its different sections.

The rest of the paper is organized as follows. In the remaining two subsections, we describe some of the related works in this area and provide a brief overview of compression techniques. This is followed by a description of the materials and methods employed to perform this review as they pertain to the Preferred Reporting Items for Systematic Reviews and Meta-Analyses (PRISMA) framework. We then proceed to disclose the results of the review by presenting our findings related to HSI compression algorithms, their classes, and their hardware platforms. Next, we discuss the comparative performance results of these algorithms and other aspects related to their hardware implementation and utilization. Before concluding this paper, we uncover the current research gaps in this area of study and provide a set of recommendations for future research. Figure 2 depicts a graphical organization of the paper to assist readers in accessing its different parts.

### 1.1. Related Work

Recent related works include a review of hyperspectral image compression algorithms published in [14]. It provides a detailed categorization of the HSI compression algorithms according to selected parameters. Another review, conducted in 2009, discusses image compression systems onboard space missions in general [15]. It covers more than 40 of these space missions planned from 1986 and up to 2010. A third study reviewing spaceborne hyperspectral missions was undertaken in 2013 with a primary focus on lossless compression type [16]. Another survey was published in 1999, dealing with lossy compression algorithms used onboard space flights by France’s space agency, known as the Centre National d’Etudes Spatiales (CNES) [17]. A review presented in [18] discusses selected topics of HSI compression for each of the three types: lossless, lossy and near-lossless compression. Babu et al. in [19] presented a review on statistical and wavelet-based compression algorithms with a focus on encoding schemes to reduce the transmission overhead. Moreover, the review by Dusselaar and Paul presents a categorization of intra-band and inter-band compression techniques of HSI [20]. It also provides an experiment to study the PSNR of selected compression algorithms. A literature survey of satellite image compression in [21] provides a comparison at the algorithm level of lossless and lossy compression types. Lossless algorithms are compared against the obtained bit rate, while the selected lossy algorithms are compared against both bit rate and signal quality.

With a focus on medical images, a survey in lossless and lossy compression algorithms is disclosed in [22]. It discusses their techniques, limitations, and compression rates. Further, a short review of some lossless image compression techniques in remote sensing applications and their implementations on FPGAs is provided in [23]. The paper includes recommendations for the development of onboard hardware-accelerated image compression and lists the advantages and disadvantages of the covered methods. Another survey on the use of FPGAs in hyperspectral remote sensing presented coverage on both technological issues and implementation aspects of HSI compression and linear unmixing techniques [24]. In this regard, the authors provide two case studies to illustrate the role of FPGAs in future spaceborne missions for Earth Observation. We present a summary of these reviews in Table 1. What distinguishes our paper is its scope and focus. We provide a systematic review of hardware-accelerated compression of hyperspectral images covering all compression types. We also emphasize multiple hardware-specific performance metrics while presenting a comparative analysis of related literature sources spanning a longer period of time.

### 1.2. Platforms for Hyperspectral Instruments

Hyperspectral instruments are integrated with different types of platforms, including spaceborne, airborne, Unmanned Aerial Vehicles (UAVs), on the ground as handheld devices or in the laboratory, and even underwater. These platforms support different spatial scales. For instance, spaceborne sensors offer a spatial resolution of 20–50 m [25]. On the other hand, airborne sensors provide less spatial resolution ranging from 0.5 m to 20 m, while miniaturized sensors can provide a corresponding resolution in the range of 1–10 cm [25]. Spaceborne sensors allow for frequent observations and wide coverage [26]. The most widely used spaceborne sensor was Hyperion, even after its shutdown in 2017 [27]. Multiple studies summarize spaceborne hyperspectral sensors and their characteristics [16,27,28].

Compared to spaceborne sensors, the airborne variety can provide higher spatial and spectral resolutions [28]. AVIRIS was the first sensor to acquire continuous narrow bands simultaneously. These spectral bands range from the visible to the Short Wave Infrared (SWIR) region of the spectrum [26]. Aircrafts follow a flight path at medium to high altitudes (20 km for AVIRIS), with high to medium spatial resolutions (20 m for AVIRIS) [27]. Hence, airborne sensors are commonly preferred when studying regional characteristics. Such platforms also offer flexibility in the acquisition process when considering the weather and solar illumination conditions [28]. In addition, sensor maintenance and adjustments can be easily conducted for such airborne sensors when compared to spaceborne ones [28]. We refer the reader to studies [27,28] for more details about airborne hyperspectral sensors. UAVs can be remotely controlled to perform autonomous flight maneuvers using an embedded autopilot. UAVs fly closer to the ground where the influence of atmospheric conditions is insignificant [29]. In addition, they have the advantage of fast deployment due to the compact sensors employed [27]. However, the low acquisition height, unstable movement, and varying illumination conditions may create challenges for geometric and radiometric corrections [25]. Details about hyperspectral sensors aboard UAVs and their respective characteristics are presented in [29].

Ground hyperspectral sensors, such as handheld or laboratory sensors, obtain numerical measurements almost in contact with the target. Therefore, they allow for more accurate readings of a given target in isolation of variable conditions. Samples can be scanned on-site or taken to a laboratory for data acquisition [27]. These samples are relatively free of “noise” and can be utilized to build spectral libraries for subsequent spectral unmixing of hyperspectral data [26]. Further details about ground hyperspectral sensors are presented in [26,27]. Another type of instrument with limited research literature is underwater hyperspectral imaging. Application domains of the underwater environment include monitoring and identification of deep-sea creatures, marine mining applications, and underwater pipeline inspection [30]. Many of the over-surface techniques do not work underwater, as the latter is more complex and dynamic. Besides, deep-sea areas cannot be imaged using passive hyperspectral imaging, and a light source is required for illumination. The study in [30] presented a survey of the major underwater hyperspectral imagers and listed a variety of underwater vehicles that may be used with those imagers. For instance, the Underwater Hyperspectral Imager (UHI) is positioned into a Remotely Operated Vehicles (ROV) for seafloor exploration [30].

### 1.3. Overview of Compression Techniques

The size of hyperspectral images can be reduced by either compression or Dimensionality Reduction (DR). Compression is concerned with preserving all captured data encoded with a reduced number of bits than the original data representation. However, DR opts for only a subset of these data according to one or more specific criteria. The main criteria for which the data subsets are selected as well as the DR main techniques, are presented in [31]. This review focuses only on compression techniques and their hardware acceleration on different computing platforms. Generally, there are two main classes of compression algorithms: lossless and lossy algorithms. Lossless compression is traditionally preferable since it preserves all information contained in the image. However, the compression ratio obtained with this compression type is limited. In contrast, lossy compression produces significantly higher compression ratios with a degradation in the image quality. A third class termed near-lossless is defined to constrain the amount of loss due to compression. An algorithm is categorized as near-lossless when the compression error is controlled below the intrinsic error of the original image, i.e., errors due to sensor calibration or atmospheric correction [32].

Typically, the compression algorithms are categorized into three main implementation methods: prediction-based, transform-based, and Vector Quantization (VQ)-based methods. The prediction-based methods depend on the correlation between adjacent pixels in hyperspectral data. The basic idea is that the difference between correlated values is encoded with fewer bits than actual values. The most basic prediction-based method is Differential Pulse Code Modulation (DPCM). The prediction-based methods have a long history and are usually recommended by the Consultative Committee for Space Data Systems (CCSDS) [33]. The transform-based methods map the spatial domain of an image into its transformation domain to decorrelate the data. Then, the coefficients with larger amplitude, or energy, are encoded with fewer codewords than coefficients with low amplitude to obtain a higher compression ratio. The conventional methods employed in transform-based compression are Principal Component Analysis (PCA), Karhunen-Loéve Transform (KLT), Discrete Cosine Transform (DCT), and Discrete Wavelet Transform (DWT). The complexity of these methods is relatively moderate and they are mainly applied for lossy compression [31]. The integer version of the transform is applied for lossless compression with limited compression ratios. Finally, the VQ-based methods quantize the data directly without decorrelation. They exploit the fact that pixels representing the same material have the same spectral information vector. VQ-based compression consists of a training step for codebook generation and a coding step where each vector is assigned to a codeword. The Generalized Lloyd Algorithm (GLA) is a common method of this type [34]. Another technique, called Self-Organizing Feature Map (SOFM), uses a neural network for codebook generation and is based on unsupervised learning. VQ-based compression can obtain higher compression ratios. However, the high number of computations needed restricts its application for real-time processing [31].

## 2. Materials and Methods

We performed a systematic search for papers covering a time span of nearly 22 years, starting from the year 2000 to part of 2021, following the guidelines presented in PRISMA framework [35].

### 2.1. Search Methodology

Journal articles and conference papers published until 15 May 2021, are collected from the following digital databases:

- IEEE Xplore,
- SpringerLink,
- Elsevier ScienceDirect,
- ACM Digital Library,
- Wiley Online Library,
- Scopus, and
- Web of Science.

The search is conducted using the query string “Hyperspectral AND Compression AND (FPGA OR GPU OR ASIC)”. To include as many papers as possible, we then searched Google Scholar for the relevant papers using the same keywords with the additional terms: “AND (satellite OR “remot* sens*” OR onboard OR spaceborne) -book -review -survey”. We limited these search results to anywhere between 45 and 46 online pages. This range appears to fulfill how far our search can go back in time. All references are then imported to Endnote and automatically scanned for duplicates. After eliminating any duplicates or multiple versions of the same paper(s) as well as removing all review papers, the remaining sources are shortlisted by screening the title, abstract, introduction, and conclusion sections of each paper.

### 2.2. Inclusion and Exclusion Criteria

To be included in the review, studies must:

1. Discuss hardware-accelerated compression algorithm(s) of remotely sensed hyperspectral images; and
2. Be journal articles or conference papers that are dated from the year 2000 to 15 May 2021.

A paper is excluded if it satisfies at least one of the following criteria:

1. The paper does not contain a hardware acceleration;
2. The paper addresses data types other than hyperspectral data;
3. The paper discusses image processing technique(s) other than compression; or
4. The paper is intended for applications other than remote sensing.

Moreover, if a relevant study is found in the reference section of any of the collected papers, it is considered for this review after fulfilling the inclusion criteria and not satisfying any of the exclusion criteria stated above.

### 2.3. Data Compilation

In addition to general information such as author name(s) and year of publication, data are extracted manually by full-text reading to perform a comparative meta-analysis, including compression type, compression algorithm, hardware architecture, programming language, hyperspectral imager, HSI dataset, scanning orders, bit depth, compression ratio, throughput, and power requirement. Bit depth is determined by “the number of bits used to define each pixel in a digital image” [36] (p. 58). Throughput is defined as the rate at which data is processed, while power is defined as the amount of electrical energy consumed per unit of time to operate a device, measured in Watts. For GPUs, if a study does not include the value of the required power, it is replaced by the manufacturer’s Thermal Design Power (TDP). TDP is related to the maximum energy generated by a hardware component or chip. For FPGAs, the required power depends on the logic configuration and the clock speed. Therefore, if it is not provided by the author(s), it is left unspecified. Further, the compression ratio is defined to be equal to the number of bits per sample before compression divided by the average number of bits after compression. Studies either provide a direct compression ratio, or alternatively, the compression rate. The unit of compression rate is bits per pixel (bpp) or bits per pixel per band (bpppb). Other missing data, such as the device specifications or information on the used hyperspectral sensors, are collected by online searching of the manufacturers’ or space agencies’ websites, respectively. Since the extracted data in various papers may be presented in different formats, conversion is carried out to standardize the results for data synthesis.

The selected papers are classified according to the compression type, compression algorithm family, computing platform, programming method, and imager by which the dataset is acquired. For the performance analysis to be meaningful, results are grouped according to the HSI datasets used in the studies. The extracted metrics are prepared for comparison using the following procedure:

- When the compression rate is given in bpp or bpppb, the compression ratio is simply calculated by dividing the bit depth of the test image by the compression rate.
- Throughput is converted to Mega Samples per second (MSps) after ascertaining the bit depth of the test image.
- Power requirement is obtained in either Watts (W) or milliWatts (mW). All power values are presented in Watts for comparative analysis.

By following the PRISMA framework and setting the inclusion and exclusion criteria a priori of the search process, we estimate that bias has been minimized in this review. However, multiple studies focus on some performance measures and neglect others, which could introduce challenges in the comparative performance analysis phase. To further reduce potential bias, extra effort is made to find the missing data when possible. For instance, to obtain the missing power requirement of a device, we resorted to consulting the information available from the device manufacturer, either from online or analog sources.

We used throughput, required power, compression ratio, and efficiency as the metrics of choice in our review because our emphasis is not on evaluating the quality of the compressed hyperspectral image per se, but more on assessing the hardware acceleration of identified compression algorithms. In addition, the use of other quality metrics for lossy compression varies across many of the collected studies. For instance, some studies use the misclassification rate, from which the classification accuracy can be obtained, as in [37,38,39]. Other studies [40,41,42,43,44,45,46,47] use the Spectral Angle Difference (SAD) while the Signal-to-Noise Ratio (SNR) is omnipresent in [44,48,49,50,51]. Further, the Peak Signal-to-Noise Ratio (PSNR) is adopted in [49,50,52,53,54,55,56,57,58,59,60,61]. PSNR is the most frequently used quality metric, yet it is more content specific. This means that when two images with different bit depth values are corrupted with the same amount of noise, the resulting PSNR values will also be different. This is a drawback since the degradation of the image quality is not caused by an external factor but by the model itself [62]. Also, the Mean Square Error (MSE) and Root MSE (RMSE) metrics are employed in [46,49,51,55,63,64,65,66] while the Normalized MSE (NMSE) is used in [56,67,68,69]. The use of normalization facilitates comparison between different datasets. However, it usually involves division by the range, which can hamper comparisons when extreme samples exist, especially for small-sized datasets. The Mean Absolute Error (MAE) metric, or Mean Absolute Deviation (MAD), is used in [46,49,51,55,65]. Finally, the work in [70] uses the percentage of retained information as an indication of signal quality.

## 3. Descriptive Analysis

**Figure 3:**
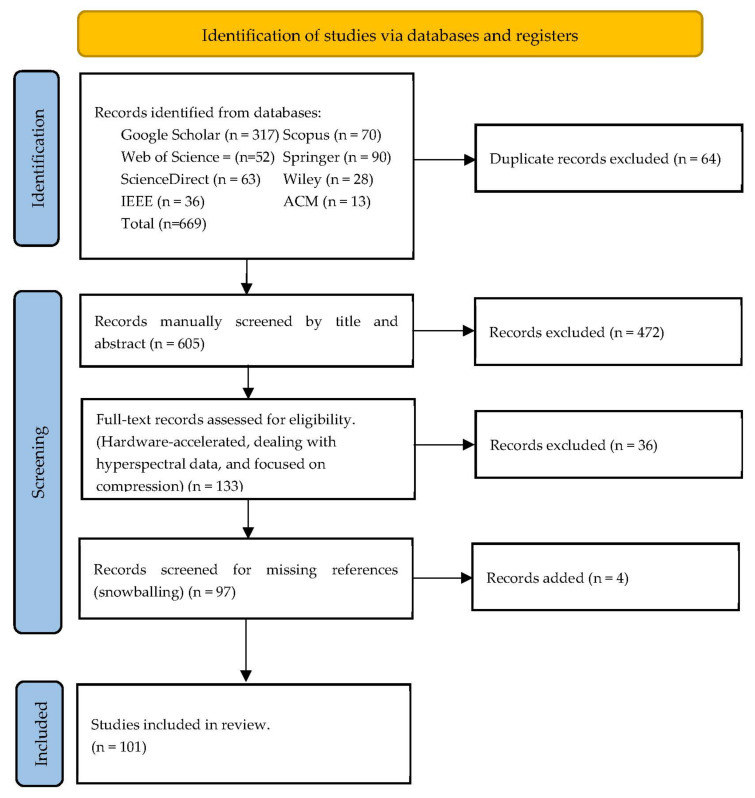
The records selection process using the PRISMA framework as applied in this review.

**Figure 4:**
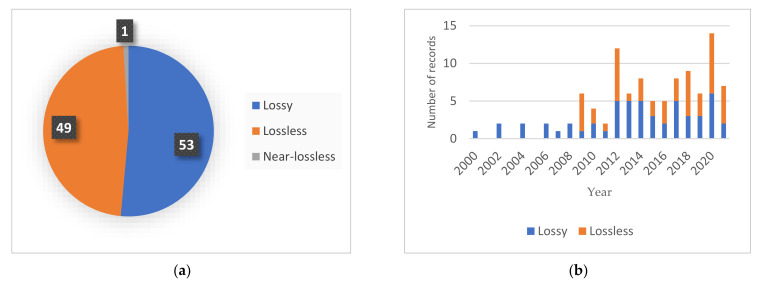
(**a**) Chart representing the number of records in different types of compression algorithms of HSI; (**b**) Temporal evolution of the distribution of number of records in each of the two major compression types (lossy and lossless) from the year 2000 to part of 2021.

A total of 699 records are collected by searching across the previously identified databases. After applying the PRISMA framework, shown in Figure 3, a total of 101 records are eventually selected for meta-analysis. Out of the 101 records, 55 are conference papers (accounting for ≈55%), and 46 are journal articles (amounting to ≈45%). The first relevant record [37] was published in 2000 and discussed an FPGA-based lossy compression algorithm for hyperspectral images by means of k-means clustering. A number of records appear to meet the inclusion criteria [71,72,73,74]. Other interesting papers worth mentioning include the works in [75,76,77]. However, each one of the latter three works did not satisfy one of the inclusion criteria. We explain in Table 2 the reasons for excluding all of these seven studies. Only one study [32] falls under the near-lossless category, and the rest are almost equally divided between lossy and lossless compression (see Figure 4a). The work in [47,78] present two types of compression and are tallied twice, one for each type. We observe that, till 2008, the early studies on hardware-accelerated compression were solely focused on lossy compression. Interest in hardware-accelerated lossless compression started to gain the attention of the research community in 2009. Then, it increased thereafter, perhaps due to the growing demand for loss-free hyperspectral images by a myriad of research and development projects for various analysis tasks. These results are displayed in Figure 4b.

In the remainder of this section, we address research question RQ1: *What are the main hardware platforms and HSI datasets used to accelerate and evaluate HSI compression algorithms in remote sensing applications?*

**Figure 5:**
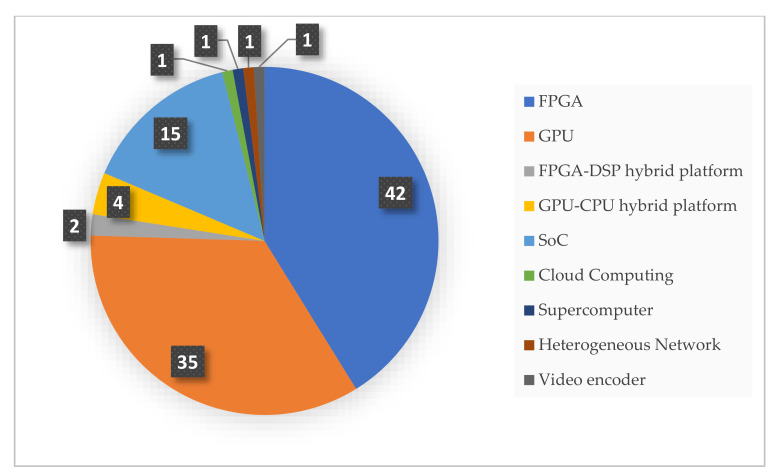
Statistics showing the number of the adopted hardware platforms for HSI compression.

The list of hardware platforms employed for HSI compression is depicted in Figure 5. Our results indicate that 42 papers used the FPGA platform, 35 studies used GPUs, and 15 employed SoCs to implement their proposed HSI compression solutions. In the remainder of the paper we use the term FPGA-based platforms to refer to the following architectures: FPGAs, SoCs, and FPGA-DSP hybrid platforms. FPGA-based platforms allow for the processing of complex computational tasks with superior performance in terms of power requirements and throughput. Further, the industry has recently made available radiation-hardened models that offer data integrity, making FPGAs the best candidate for small-satellite missions [79]. While GPUs show remarkable performance and flexibility, they are characterized by high-power requirements and a lack of radiation tolerance [80,81]. The work in [82] uses both FPGA and GPU implementations and is counted once for each category. Hybrid GPU-CPU solutions are also adopted to improve the total performance by utilizing features of different hardware architectures, as presented in [49,69,83,84]. In addition, records are found that employ parallel architectures such as supercomputers [85], cloud computing platforms [46], and heterogeneous networks of workstations [86]. Although not suitable for onboard compression, the parallel computing-based techniques presented in these studies can be exploited and migrated onto portable machines. Finally, video encoders are proposed in [44] for compressing hyperspectral images as an attempt to reuse existing solutions. Papers employing only CPU-based computing platforms are excluded as this review is focused on hardware-accelerated compression employing high-performance architectures.

**Figure 6:**
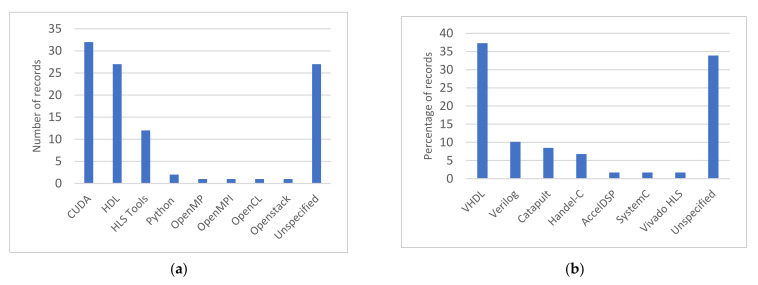
(**a**) Distribution of the collected papers in terms of the programming methods (languages and tools) used for hardware-accelerated compression of HSI; (**b**) Distribution of the records as percentages in terms of the employed programming methods for FPGA-based platforms used in the acceleration of HSI compression.

In terms of implementation, CUDA (Compute Unified Device Architecture) is mainly used for programming GPUs. However, we found one study that employs Python for programming GPUs [83]. Python is also used in [85] with the PARAM-SHIVAY supercomputer, where parallel programming is implemented using a preinstalled multiprocessing library. FPGA-based platforms are usually configured using Hardware Description Languages (HDL) such as VHDL or Verilog HDL. In this regard, Figure 6a shows the distribution of the selected studies according to the employed programming method. We note that when more than one programming method is found, the record is counted once for each method. Figure 6b shows the distribution of HDLs and High-Level Synthesis (HLS) tools when hardware-accelerated compression for HSI used FPGA-based platforms. We observe that approximately 46% of these compression solutions are implemented using HDLs and about 20% are implemented by means of HLS tools. Such tools include Handel-C [38,41,42,87], CatapultC [88,89,90,91,92], SystemC [10], Vivado HLS [92] and AccelDSP synthesis tool [93]. The remaining 34% of the compression solutions on FPGA-based platforms did not specify their respective implementation method. HLS tools are used to avoid the complexity of programming in low-level languages and to speed-up the implementation task. However, they generate less efficient code than direct coding with HDLs [94].

**Figure 7:**
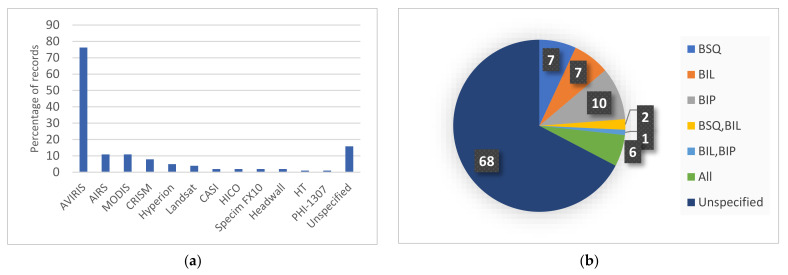
(**a**) Percentage of studies employing each of the different hyperspectral imagers’ datasets. Note that the total number of datasets used is more than the number of records because some of these validated their results using more than one dataset; (**b**) Chart showing the number of studies using each of the scanning order types in HSI compression.

We further observed that about 76% of the studies benchmarked their systems using the AVIRIS datasets, especially the Cuprite scene, as shown in Figure 7a. Images obtained by both AIRS and the Moderate Resolution Imaging Spectroradiometer (MODIS) are each used to validate nearly 11% of the proposed hardware-accelerated solutions. Other spectral imagers are also utilized, such as the Compact Reconnaissance Imaging Spectrometer for Mars (CRISM), Hyperion, Landsat, the Compact Airborne Spectrographic Imager (CASI), and the Hyperspectral Imager for the Coastal Ocean (HICO). Specific hyperspectral cameras are employed as well, such as Specim FX10 [49,51] and PHI-1307 [95], the latter being an imager developed by the Shanghai Institute of Technical Physics. It is noted here that the total number of datasets used is more than the number of records because some papers validated their results using more than one dataset. Rarely used imagers or synthetic images are difficult to incorporate in the performance analysis. Nonetheless, the main idea behind their proposed compression systems is presented herein.

Depending on the scanner type, HSI samples can be arranged in three different formats: Band Sequential (BSQ) by snapshot scanners, Band Interleaved by Pixel (BIP) for whiskbroom scanners, and Band Interleaved by Line (BIL) for pushbroom scanners. For onboard compression, it is assumed that the compression process takes place in the same order as that of the samples’ arrival. Despite the scanner type, the test hypercube can be fed to the compression system in the order that represents the best fit for the compression algorithm. However, for onboard real-time compression, the scanning format used must match the acquisition order of the hyperspectral data. Studies usually adopt the format according to its suitability for the proposed algorithm. We also reviewed studies that benchmarked their compression systems using all three scanning orders [10,82,96,97,98,99]. The distribution of the selected papers based on the adopted scanning format is presented in Figure 7b. Ten papers used the BIP format, while the BSQ and BIL formats were reported in seven papers each. These three formats appear to be nearly equally used in the validation of the compression results of HSI. A total of 68 studies left the type of scanning format unspecified.

## 4. Hardware-Accelerated Compression Algorithms of HSI

This section discusses the hardware-accelerated compression algorithms based on their class and thus addresses the second research question RQ2: *What are the different HSI compression algorithms and their classes that are accelerated in hardware?*

**Figure 8:**
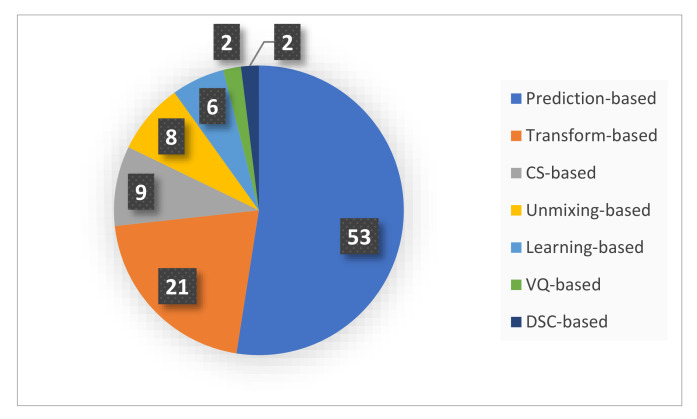
Chart showing the number of studies according to the compression algorithm class on which they are based.

**Figure 9:**
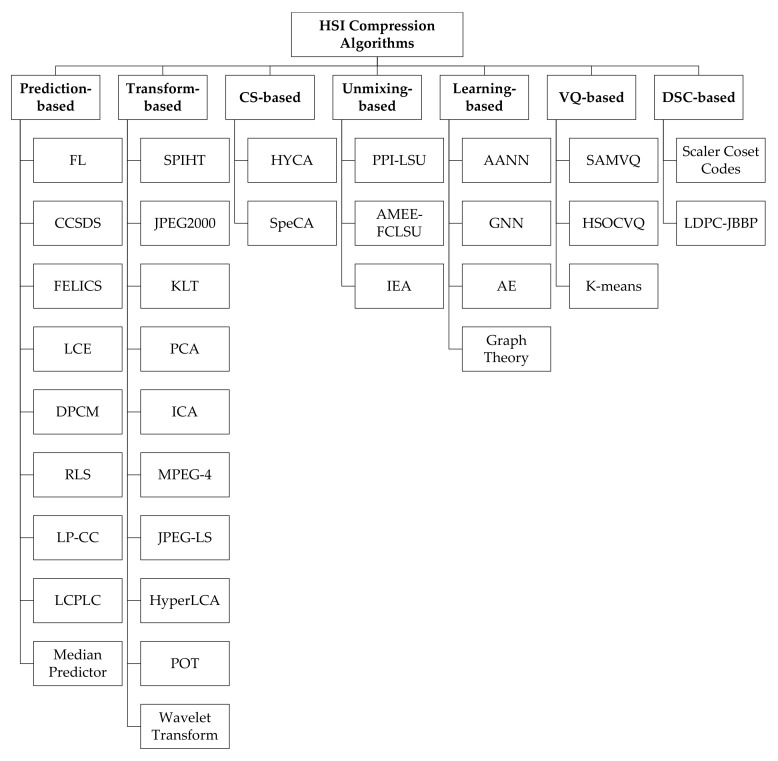
Taxonomy of HSI compression techniques generated by this review.

The included research papers in this review are categorized into seven classes according to the algorithm family: prediction-based, transform-based, VQ-based, unmixing-based, learning-based, Distributed Source Coding-based (DSC), and Compressive Sensing (CS) methods. Figure 8 shows the distribution of the studies according to the algorithm class. The majority of the reviewed studies (53) are focused on prediction-based algorithms. The transform-based methods are found in 21 of the studies, whereas spectral unmixing methods are covered in eight of the studies, with the most recent being conducted in 2012 [45,64]. Furthermore, hardware-accelerated compression of hyperspectral images using learning-based techniques is found in six of the studies employing different types of Neural Networks (NN) and Autoencoders (AE). Compressive sensing has caught the attention of more researchers as nine of the studies fall under the CS category. Finally, only two relevant studies each for the categories of DSC-based [100,101] and VQ-based [32,37] were identified in this review. Figure 9 shows a hierarchical categorization of the compression algorithms according to the algorithm class. It represents a taxonomy of various compression algorithms covered by this review.

### 4.1. Prediction-Based Algorithms

**Figure 10:**
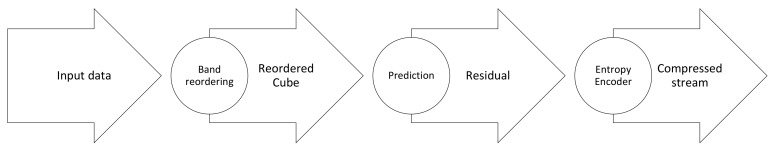
Main steps involved in prediction-based compression of HSI.

A prediction-based compression algorithm typically depends on the correlation between adjacent pixels in hyperspectral data. The basic idea is that the differences between correlated values are encoded with fewer bits than the actual values [31]. In this class of algorithms, compression generally consists of three main steps, as depicted in Figure 10. First, band reordering is applied to improve the obtained compression ratio. Second, the predicted values are generated across the spectral or spatial dimensions, as well as across all three dimensions of the datacube. Finally, the differences between the original and predicted values are passed to the entropy encoder to generate the compressed stream [102]. Band reordering increases the computational complexity. Therefore, it is usually computed on the ground using pre-acquired samples and then uploaded to the satellite as a Look-Up Table (LUT). Band reordering depends on the sensor and the scene, so the benefit from offline band reordering is reasonable in some cases and negligible in others. For each of the compression algorithms in this class having been accelerated in hardware, we provide next some of the related details that are specific to this review.

#### 4.1.1. Fast Lossless

References [103,104,105,106,107,108,109] cover Fast Lossless (FL). FL is a prediction-based algorithm developed by Jet Propulsion Laboratory (JPL). It uses the Sign algorithm, a low-complexity variation of the Least Mean-Square (LMS), for adaptive filtering. Samples are computed by linear prediction and corrected by subtracting the local mean. Then, the differences between the predictive and the actual samples are encoded. The hardware acceleration of Fast Lossless has dramatically improved the performance of the algorithm in [103] using Xilinx Virtex-IV LX160 FPGA. Uncalibrated AVIRIS images show a compression ratio of 4:1 and a throughput of 33 MSps with a power requirement of 1.27 Watts. A modified FL, designed for images acquired by pushbroom imagers, was presented in [104]. Results show significant improvement when the local mean equals the previous sample in the same cross-track position and the same band. The modified algorithm boosts the throughput up to 58 MSps with similar compression ratios and within the same power constraints. On the other hand, a CUDA implementation of the algorithm targeting Nvidia GeForce GTX 580 GPU produces only 44.85 MSps [105]. Further modification of Fast Lossless is proposed to increase the compression ratio up to 5.5:1 [106]. However, this improvement comes at the expense of reducing the system throughput to 40 MSps. The implementation targets Xilinx Virtex-5 SX50T and Virtex-6 LX240T FPGAs with a required power of 700 mW. Combined with a radiation hardening technique, a pure software implementation of Fast Lossless produces 2.58 MSps running on FPGAs integrated with PowerPC 405 processors [107]. An enhanced speedup of 11.28 is achieved compared to the software implementation when migrating the key functions of the predictor into the FPGA fabric of Xilinx Virtex-4 FX60 [108]. The extended version of FL, namely FL Extended (FLEX), combines lossless and lossy compressions. The lossless part represents the CCSDS 123.0-B-1 standard for lossless multispectral and hyperspectral compression. The algorithm uses adaptive filtering and exploits redundancy in all three dimensions, spatially and spectrally. Three IP cores of FLEX are integrated into the Zynq SoC device producing a compression rate of 70 frames per second (Fps) benchmarked with the AVIRIS Hawaii hyperspectral image [109].

#### 4.1.2. Fast Efficient and Lossless Image Compression System

Fast Efficient and Lossless Image Compression System (FELICS) is a compression algorithm that performs significantly faster than the lossless JPEG algorithms, JPEG2000 and JPEG-LS. FELICS is adopted for further improvement in the prediction and encoding phases [110]. The improved predictor uses four reference pixels instead of two in the original FELICS, which yields better coding efficiency. Besides, conditional branches are reduced for improved computational efficiency. The predictor also reduces memory access to a single pass, instead of two in the original FELICS, by reusing the preceding pixels for predictions. For the encoding phase, FELICS algorithms accumulate the magnitude of prediction errors which requires oversized lookup tables. The improved method accumulates only the optimal parameters of recent prediction errors, which allow for smaller lookup tables. Results show a throughput of 30 MSps at the expense of a minor increase in the compressed data size compared to the two lossless JPEG algorithms. Compression ratios of 1.7 and 2.7 are obtained using AVIRIS Jasper Ridge and Cuprite images, respectively [111].

#### 4.1.3. Edge Detectors

Edge detection-based algorithms are covered in references [93,95,112,113,114,115]. Edge detectors are image processing methods used to identify discontinuities in the image in terms of changes in brightness. In data compression, edge detectors are useful in the extraction of important image features. For instance, the Gradient-Adjusted Prediction (GAP) is a nonlinear predictor that weighs the adjacent pixels according to the gradient of the image to detect the magnitude and orientation of edges in the test image [112]. A lossless compression algorithm that employs GAP is optimized for low complexity and low power requirement [93]. The algorithm uses Vertical Scanning (VS) to process the image blocks and GAP to predict the current pixel. vs. is adopted to support the multidimensional prediction of independent regions. Therefore, GAP can be carried out using the current and the previous bands. Finally, entropy coding is performed using Extended Rice, a simplified version of Golomb codes, optimized by means of quantization. The proposed design is benchmarked using AVIRIS Cuprite on Virtex-5 FPGA. For efficient use of the resources, the 18 × 18 multipliers are replaced with a multiplier-free design as the quantization procedure reduces multiplication to 3-bit operations. Results show a compression ratio of 2.8 and throughput of 210 MSps with a required power of 573 mW.

The Median Edge Detector (MED) is a simple predictor that selects one of three predictions based on whether the window being processed represents a smooth area, a vertical or horizontal edge [113]. GAP is combined with MED, used in JPEG-LS, to improve the accuracy of the predictor [95]. Using Huffman coding, the resulting prediction errors are encoded into variable-length codewords. However, FPGA registers can only produce fixed-length sequences. Therefore, additional zeros are added to the codewords to reach a fixed length of 20 bits limiting the compression ratio to 2.3. The compression method is implemented on Xilinx Spartan-3E FPGA using Verilog HDL. The proposed method presents comparable performance to JPEG-LS with reduced complexity. The median predictor is also employed in [114] for the intra-band prediction. Then, the inter-band prediction follows, where the initial prediction is calculated and passed to a multi-lookup table structure to produce the final prediction. Index quantization is applied to reduce the size of the lookup tables. Finally, entropy encoding is carried out using adaptive arithmetic coding. A compression ratio of 3.74 is achieved at a throughput of 16.5 MSps utilizing Xilinx Spartan3 FPGA with an embedded ARM processor. Similarly, the median prediction is employed for intra-band prediction in [115]. However, the inter-band prediction is carried out using a hybrid predictor that combines linear prediction and context prediction. The last stage is entropy coding of the residual data utilizing Huffman coding. This approach has achieved a compression ratio of 3.28 with a required power of 1194 mW.

#### 4.1.4. Lossy Compression of ExoMars

HSI compression based on Lossy Compression of ExoMars (LCE) is disclosed in references [55,88,89,90,91,116,117]. LCE is an algorithm designed for onboard image compression for the European Space Agency (ESA) ExoMars mission. It consists of four phases: prediction, rate-distortion optimization, quantization, and entropy coding using Golomb codes. Initially, the algorithm is implemented using ANSI C language for sequential execution on a CPU. Parallel CUDA implementation is proposed by creating two kernels; one for the first three phases and another for the entropy coding [116]. Results show a throughput of 12 MSps using the Nvidia Tesla C2075 GPU. The implementation is evaluated using test images from multiple imagers, AIRS, MODIS, and AVIRIS. An improved CUDA implementation is proposed with an extra kernel to perform bit packing [117]. A notable throughput that exceeds 100 MSps using the same GPU architecture is achieved. Further investigation on the impact of varying step sizes of the quantization phase is found in [55]. LCE is also implemented on the FPGA platform by means of HLS tools, namely CatapultC [88]. The source code is first prepared for synthesis. Then, data types are converted to algorithmic C, and dynamic memory allocation is replaced by fixed data size. The compiled code was executed on the Virtex-5 FPGA and yielded a throughput of 19 MSps. Improved throughput of 26 MSps is achieved in [89]. Instead of generating a Register-Transfer Level (RTL) design directly from the source code, it first splits into independent modules where connections and control logic are manually written. The LCE algorithm is also accelerated using the anti-fuse Microsemi RTAX2000 FPGA at lower throughput of 5–6 MSps and with only 400 mW of power [90]. These studies on LCE algorithm utilizing different architectures are collectively analyzed in [91].

#### 4.1.5. Clustered Differential Pulse Code Modulation

The Clustered Differential Pulse Code Modulation (C-DPCM) is a prediction-based algorithm that clusters similar spectra of the input hyperspectral image into classes. Then, the current band of each class is predicted from the previous band using linear prediction [118,119]. A lossless compression by means of C-DPCM is combined with the removal of spectral outliers in [118]. The proposed algorithm consists of three steps: clustering, prediction and coding. First, linear regression produces the predicted values for each cluster. To minimize the residual, the predicted values are then used to remove spectral outliers in each cluster. The remaining spectral vectors produce the final prediction values by performing a second round of linear regression. Finally, the residuals, obtained by the difference between the original and the predicted image, are entropy encoded using arithmetic coding. A GPU implementation of C-DPCM aims to enhance the aggregate throughput by employing multiple optimization strategies in [119]. One of these strategies uses shared memory and registers, another employs a multi-stream technique, and a third by using a multi-GPU platform.

#### 4.1.6. Low Complexity Predictive Lossy Compression

Low Complexity Predictive Lossy Compression (LCPLC) is an algorithm based on prediction, uniform threshold quantization, and rate-distortion optimization. A hardware acceleration of LCPLC that employs pipelining is proposed in [60]. Two levels of pipelining are introduced in this architecture. High-level pipelining across modules and another level of pipelining within each individual module. The proposed approach maintained a throughput of 162 MSps with a power requirement of less than 1 W.

#### 4.1.7. Recursive Least Squares

References [85,120] deal with Recursive Least Squares (RLS). RLS is an adaptive filtering algorithm that recursively finds the coefficients to minimize the least square estimation of the filter weight vector. An optimized RLS is implemented using CUDA on Nvidia Kepler GTX 690 GPU based on the optimal number of bands to improve the bit rate [120]. The basic idea behind the optimized algorithm is to spread the spectral information to the neighboring pixels until a stable global state of the image is reached. Three variations of RLS are explained in [85]: Conventional RLS (CRLS), RLS Adaptive Length Prediction (RLS-ALP), and Fast-RLS-ALP. CRLS is similar to the original RLS except for the context window size in the spatial decorrelation phase, where 24 instead of four neighboring pixels are used. RLS-ALP produces optimal results, yet it is more time-consuming as the algorithm runs multiple times while changing the prediction length (number of bands) in order to find the optimal length. Fast-RLS-ALP addresses the time complexity issue by replacing the multiplication with an append operation when calculating the weight matrix. The complexity is reduced from *O(p*^2^*)* to *O(p)*, where *p* is the number of bands. The optimal prediction is reached when the number of bands is equal to 28 with a negligible impact of the context window size.

#### 4.1.8. Linear Prediction with Constant Coefficients

Linear Prediction with Constant Coefficients (LP-CC) is initially proposed for ultraspectral sounder data in [121] and validated with AIRS sounder data. AIRS collects as many as 240 granules (sounder-generated datacubes) per day. Each granule consists of 135 lines, 90 footprints and 2378 spectral channels. Radiance data is converted into a 16-bit unsigned integer producing a total size of around 110 Gigabits [102]. In LP-CC, the coefficients are constant as they are computed for a randomly preselected set of granules and then used to compress all other granules. A CUDA implementation of the parallel version of the algorithm in [122] shows a speedup of 30 times compared to the sequential implementation.

#### 4.1.9. Consultative Committee for Space Data Systems Standard

Most of the studies in the prediction-based class of HSI compression algorithms follow the CCSDS 123 standard and are treated in [10,50,79,80,81,82,92,96,97,98,99,123,124,125,126,127,128,129,130,131,132,133,134,135,136,137,138,139]. This compression standard is designated for lossless compression by the consultative committee for space data systems. It is a causal algorithm that uses only previously processed pixels for the current prediction. First, the residual is calculated using *N* neighboring pixels. Similarly, pixels at the same location are calculated across *P* previous bands. The resulting residual vector is then multiplied by the weight vector that is updated in each iteration according to prediction error. The outcome of this multiplication is a scaler value. The final step is mapping the scaled prediction residuals.

The suitability of two hardware architectures is investigated for real-time compression based on CCSDS 123.0-B-1 [123]. An OpenMP implementation of the algorithm on the multicore Intel Core i7-2760QM processor yields a throughput of 128 MSps. Further speed-up is achieved using Nvidia GeForce 560M GTX GPU at a throughput of 322 MSps, whereby the data dependency inherited in the algorithm is removed by employing suitable buffering. A compression ratio of 5.3 is achieved for the AVIRIS Hawaii scene as a test image. A near-lossless compression is obtained by adding a quantization stage to the lossless compression standard of CCSDS 123 in [124]. An FPGA implementation of the compressor reaches 20 MSps targeting a radiation-hardened Xilinx Virtex-5. Another implementation that reformulated the CCSDS 123 algorithm at the hardware level has achieved a throughput of 55.4 MSps [125]. It was benchmarked with images acquired by AVIRIS, MODIS and CRISM over the space-grade Virtex-5 FX130 FPGA. A CUDA implementation of the CCSDS 123 algorithm employs tiling to present an additional level of parallelism is discussed in [126]. The on-chip memory is utilized to cache any intermediate compression variables. Using a platform that combines an Intel Core i5-3470 processor and GeForce GTX 750Ti GPU, a throughput of 301 MSps is achieved at a compression ratio of 4:1. Moreover, an error-resilient model using a low-power embedded GPU, the Jetson TX1, achieved a reduction in throughput up to 3 times compared to GeForce GTX 750Ti [127].

A hardware acceleration named HyLoc, based on CCSDS 123, is implemented over multiple FPGAs: RTAX2000S, Virtex-5, and Virtex-IV LX160 [80]. A throughput of 11.3 MSps and a compression ratio of 3.4 are achieved. In the hardware implementation of HyLoc, the current pixel to be compressed and the previously processed neighbors are stored in FIFO (first in, first out) buffers at the compressor’s input to reduce the number of accesses to the external memory and speedup the compression of the subsequent pixels. The work in [128] is another study that increased the throughput of HyLoc to a value of 20 MSps. Additional levels of parallelism are identified as there is no dependency between the update of each weight value, as well as the weight update block and prediction residual mapping. This allows for these independent calculations to be carried out in parallel. Multiple aspects of the HyLoc algorithm have been improved in [10], including an enhanced throughput and the ability to process multiple data formats: BSQ, BIP and BIL. The improved algorithm, named SHyLoc, combines two standards, with the CCSDS 121 performing entropy coding of the outcomes obtained by using CCSDS 123. The algorithm is designed and verified utilizing SystemC for the Electronic System-Level (ESL) modeling and Transaction-Level Modeling (TLM). In [97], the VHDL implementation of SHyLoc is presented. Synthesis results on Virtex-5 FX130 show a throughput that exceeds 100 MSps for multiple imagers’ datasets. An enhanced version of SHyLoc, named SHyLoc 2.0 in presented in [98]. It added new features to improve the algorithm performance. Such added features include the unit-delay predictor defined by the CCSDS 121.0-B-2 standard to enhance the entropy coder performance and burst transactions to communicate with an external memory on the CCSDS 123-IP. This resulted in improving the system’s throughput reaching a value of 150 MSps. Protected versions of SHyLoc by means of Dual Modular Redundancy (DMR) and Triple Modular Redundancy (TMR) are evaluated in [99]. Results show that DMR requires half the power of TMR, yet they both provide similar protection coverage.

ARTICo3 is a framework for multi-accelerator design and management. It provides three components: FPGA-based processing architecture, an automated toolchain to implement on a multicore system, and runtime management for FPGA reconfiguration and parallel execution [129]. ARTICo3 is employed in [130] to incorporate 16 HyLoc compressors that operate in single instruction multiple data (SIMD)-like fashion and are managed by ARTICo3 for dynamic partial reconfiguration. The adaptation is supported at run time by switching the number of hardware accelerators for performance-power tradeoffs. A throughput of 67 MSps is achieved at a compression ratio that ranges between 3.2 and 4. ARTICo3 is also employed in [50] to deploy a lossy extension of the CCSDS 123 lossless standard. A bit rate control stage is used in an attempt to increase the compression ratio without compromising the image quality. However, the resulting system throughput is limited to only 1.7 MSps.

A CCSDS 123 encoder is implemented utilizing a heterogeneous computing platform for low power requirement [131,132]. The implementation on Jetson TX2 GPU, equipped with an ARM Cortex-A57 and Denver 2 processors, provides a throughput of 97 MSps with only 5 W of required power. Sample splitting, being the most time-consuming task of the encoder, is executed on the GPU. The two-core Denver 2 and four-core ARM processors work concurrently to process the samples. Finally, concatenation takes place using the Denver 2 processor. Three variations of Jetson architectures are evaluated in [133]: Nano, TX2, and AGX Xavier. The latter obtained the highest throughput, reaching up to 418.7 MSps while encoding the bands sequentially at an overall power requirement of 11 W.

The real-time performance of a CCSDS 123 hardware acceleration is validated on the Virtex-4 and Virtex-7 FPGAs [96]. Instead of external random-access memory (RAM), the implementation utilizes the board memory as a cache for the sensor’s data and a buffer for storing temporal data needed for the compression. A comprehensive study that extends the evaluation to Virtex-5 FPGA and two GPU platforms is presented in [82]. Results demonstrate that the FPGA platform offers the best tradeoff considering both throughput and power requirement. In addition, real-time performance of CCSDS 123 is achieved in [134] by utilizing BIP ordering. Unlike BSQ and BIL orderings, BIP does not require the prediction of the current sample to be complete before the prediction of the next sample begins. With a Zynq-7020 FPGA operating at 147 MHz, a throughput of 147 MSps is achieved, producing one sample per clock cycle.

An FPGA implementation of the CCSDS 123 algorithm based on the principles of C-slow retiming is proposed in [81]. C-slow retiming allows pipelining of the critical path. Each register is replaced with C registers to enable multiple streams of computations, and retiming optimizes the balance of these registers by placing them forward or backward. This process, utilizing the task-level parallelism inherited in the algorithm, increases the aggregate throughput of the design to reach 213 MSps. A higher throughput reaching up to 750 MSps is accomplished in [79] while maintaining a required power of 515 mW. The proposed solution employed advance routing with shifting and delay operations. Besides, the packing operation of the resulting variable length codewords is done concurrently. Segment-level parallelism is also employed in CCSDS 123 to increase the resilience of the compression system to errors [135]. The hyperspectral cube is partitioned into segments and compressed in parallel using five core compressors implemented on Zynq-7045 FPGA fabric and controlled by a software scheduler hosted in a CPU. A throughput of 1387 MSps is achieved with a power requirement of 8.2 Watts. The use of HLS tools is investigated in [92] for CCSDS 123-based compression on FPGA architectures. CatapultC, as an HLS tool, is technology independent and can be used with FPGAs from different vendors. However, it was reported that Vivado HLS outperforms Mentor Graphics’ CatapultC when Xilinx FPGAs are employed.

CCSDS 123.0-B-1 is implemented using an FPGA for a 3U Cubesat, yielding a compression ratio of four [136]. NOR-based flash memory is shared between the On-Board Computer (OBC) and the payload, i.e., the hyperspectral imager. The satellite enters the compression mode when the shared memory has uncompressed data, and the system has enough power for compression. In the sun-tracking mode, the power is expected to increase, therefore, compression can take place simultaneously when enough power budget is reached. A 3U Cubesat is also the target platform in [137], where CCSDS 123.0-B-1 is implemented using Verilog HDL on a development FPGA board by Digilent named Zedboard. In addition, an FPGA implementation of CCSDS 123.0-B-1 is accelerated by adopting two clocks to solve the weight update feedback delay [138,139]. Here, a fast clock is dedicated to updating the weight coefficients, while the slow clock is used to calculate the predicted values. Block-based compression is also adopted to constrain the error propagation caused by the compression error of pixels. In Table 3. below, we provide a summary of the collected studies on prediction-based compression algorithms of remotely sensed hyperspectral images used on different hardware accelerators. In addition to the name of the compression algorithm, we include the hardware platform and programming method used for implementation.

### 4.2. Transform-Based Algorithms

**Figure 11:**
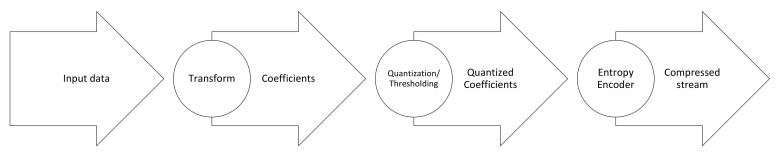
Main steps in the transform-based compression class of algorithms.

The basic idea behind this class of algorithms is to map the spatial domain of an image into its transformation domain. Then, the coefficients with larger amplitude, or energy, are encoded with fewer codewords than coefficients with low amplitude to obtain higher compression ratios. Transform-based algorithms are mainly applied for lossy compression [31] and have relatively moderate complexity. In particular, transform-based compression does not require band reordering. The transform function is first applied to generate the transform coefficients. For lossless compression, the transform function should be reversible to avoid data loss. Then, the transform coefficients are decorrelated to remove redundancy. Finally, the output coefficients are passed to the entropy encoder to generate the compressed stream. This process is depicted in Figure 11. It is worthy to note here that transform-based methods are more successful with lossy compression as the integer form of the transform limits its ability to decorrelate the data being processed. We next describe the compression algorithms that belong to this class and their respective hardware accelerations.

#### 4.2.1. Set Partitioning in Hierarchical Trees

Set Partitioning in Hierarchical Trees (SPIHT) is a wavelet-based compression algorithm. It is a progressive algorithm where the critical wavelet coefficients are encoded and transmitted first. The receiver side then performs inverse transform on the decoded coefficients to progressively refine the constructed image. SPIHT is suitable for parallelism on FPGAs as real-number coefficients are represented with multiple fixed-point formats according to the wavelet level [52]. The fixed order of the transmitted coefficients is also imposed to address the sequential nature of dynamic coefficients ordering in the algorithm. The fixed-order SPIHT significantly increases the throughput of the encoder at the expense of minor PSNR degradation. The algorithm is implemented using VHDL on the WildStar FPGA processor board populated with three Xilinx Virtex 2000E FPGAs. The obtained throughput of the system is 50 MSps using 16-bpp images.

Linear prediction precedes SPIHT as a preprocessor to utilize data correlation across bands. The prediction increases the compression ratio from 8:1 to 40:1. However, the predictor requires access to the decoded band used for prediction, which complicates the design in hardware. Miguel et al. in [63] proposed a predictor that avoids decoding and uses only full bit-planes of the wavelet transform, utilizing the fact that the transform step of SPIHT requires much less time than the bit-plane coding step. The proposed solution is benchmarked using AVIRIS Cuprite and designed to run on a two-FPGA platform.

#### 4.2.2. JPEG2000 and JPEG-LS

References [40,47,53,54,141,142,143] cover compression algorithms based on the JPEG2000 and JPEG-LS, with the first six employing the former and the last reference using the latter. The JPEG2000 standard offers two compression modes, lossy and lossless. The first step of the algorithm is to transform the RGB color space into either YCbCr for lossy compression or YUV for lossless compression. After color transformation, the image is divided into tiles, where each tile is encoded separately. Tiling is advantageous for the decoder as it reduces memory requirements. However, tiling can also create blocking artifacts in the image, similar to DCT-based JPEG. Next, DWT is applied for each tile, and the integer transform is used for lossless compression mode. In the case of lossy compression, quantization is then performed to increase the compression ratio. Finally, the resulting wavelet coefficients are grouped into code-blocks encoded separately by the Embedded Block Coding with Optimal Truncation (EBCOT) algorithm.

Compression of hyperspectral images using anomaly detection is validated in [40] by Cook and Harsanyi in 2002. Since they are compressed differently, the anomalous pixels are separated from the dominant pixels. Further compression is applied to spatial and spectral data employing the wavelet transform. The approach is benchmarked with Cuprite and Coleambally scenes acquired by the Hyperion sensor with a compression ratio of 100:1. However, the observed changes in spectral features and angular differences might affect obtained results. The proposed hardware architecture integrates FPGA and DSP technology to meet the compression requirements. In 2004, results were updated to recommend JPEG2000 for the wavelet-based transform and limit the compression ratio to 25:1 to maintain high-quality images [141]. In another work, JPEG2000 is combined with 2D-wavelet transform to compress hyperspectral images [53]. The wavelet-transform first decorrelates spatial information for each band. The decorrelated bands are then compressed using JPEG2000 at a compression ratio of 4:1. The decorrelation step is implemented on Xilinx Virtex-4 FPGA, where JPEG2000 is implemented on Xilinx Virtex-II Pro FPGA.

JPEG2000 is also employed to compress hyperspectral images produced by a Hadamard Transform (HT) spectrometer [54]. Images obtained by such a spectrometer have high SNR leading to higher compression ratios. The compression system has four main components: Universal Asynchronous Receiver-Transmitter (UART) for data transmission, static RAM as a data buffer, a designated JPEG2000 codec chip (ADV212), and FPGA to control the bus and call IP core. The codec chip can process images at a throughput of 65 MSps and a compression ratio of 8:1. Further, GPU implementations of JPEG2000 show real-time results at a compression ratio of 2:1 for both lossy and lossless modes [47]. The impact of the lossy mode on spectral unmixing is investigated in the study. Using AVIRIS Cuprite as a test image, results show that spectral similarity is maintained within a compression ratio of 13:1. The work in [142] attempts to reach real-time performance for lossy compression by applying extensive pipelining to the most consuming part of JPEG2000 when combined with PCA. In this regard, the Bit-Plane Coder (BPC) accelerates the execution time by concurrently processing bits in groups of four. Then, buffering and a system of FIFOs are incorporated to keep up with the speed requirements in feeding the BPC output to the arithmetic coder. A VHDL implementation of the proposed approach on Xilinx Virtex-7 FPGA, benchmarked with the AVIRIS dataset, provides a throughput of 72 MSps.

JPEG-LS is a lossless compression standard that consists of two main parts: context modeling and run-length coding. Context modeling imposes spatial dependency between adjacent pixels, and run-length coding is processed sequentially. In [143], A CUDA implementation of the algorithm on Nvidia GTX480 GPU using a block-based strategy results in a speedup of 26 compared to the corresponding CPU code. Because smaller block sizes have a negative impact on the compression ratio and larger block sizes reduce the degree of parallelism, they were maintained at 64 × 64 to balance these two effects.

#### 4.2.3. Video Encoder

The feasibility of H2.64/AVC video encoding standard is explored in [44] for lossy hyperspectral image compression. Validated by the accuracy of spectral unmixing, the encoder can provide high compression ratios reaching 16:1 using AVIRIS images for benchmarking. This work is intended to facilitate the future design of new architectures for HSI compression using available IP cores and related hardware components already proposed for the implementation of H.264/AVC codec on FPGAs or GPUs.

#### 4.2.4. Karhunen-Loéve Transform

KLT is an orthogonal linear transform applied to decorrelate bands and construct more compressible data. KLT requires intensive computations for the covariance matrix and eigenvector evaluation [78,144,145]. In [144], the implementation of this transform is accelerated using a low-power SoC that incorporates a flash-based FPGA and ARM Cortex M-3 microcontroller. The most consuming operations are assigned to the FPGA fabric, and the less frequent operation and task scheduling are assigned to the embedded processor. An acceleration method is suggested in [78] using a Matrix Reduction Technique (MRT) that allows for eigenvectors to be partially computed before the completion of all eigenvectors. This overlap creates an extra level of parallelism that becomes more significant with the increasing number of bands.

Pairwise Orthogonal Transform (POT) is an approximation of the KLT algorithm for spectral decorrelation of hyperspectral images. An FPGA implementation of POT by Santos et al. in [145] validates the reduced complexity of the algorithm on RTAX2000S-DSP. POT outperforms the discrete wavelet transform, although not reaching the coding performance of KLT. To complete the full compression engine, POT is tailed with CCSDS 122.0, a 2D compressor that yields 60 MSps. However, the compression engine is limited by POT to a throughput of only 12.5–18.4 MSps.

#### 4.2.5. Discrete Wavelet Transform

The DWT is a technique for multiresolution image analysis. It can also be used for compression when retaining only a few coefficients after applying the said transform. A compression algorithm based on Region Of Interest (ROI) is implemented using CUDA on an Nvidia GeForce GTX 750 Ti GPU [66]. First, the samples are clustered employing the K-Means algorithm. Then, PCA and 2D-DWT are combined for spectral and spatial decorrelation, respectively. This is followed by applying the Uniform Scalar Dead-Zone Quantization (USDZQ) before entropy encoding is performed by means of arithmetic coding. An HSI of a harbor area, captured by AVIRIS sensors, was used to test the parallel and sequential implementations. A parallel speedup of 3.21 times is achieved when compared to a CPU implementation.

#### 4.2.6. Component Analysis

Variations of Component Analysis (CA) are used for compression in [70,84,146]. In particular, hyperspectral image compression by means of dimensionality reduction is proposed in [146] employing Fast Independent Component Analysis (FastICA). FastICA consists of covariance matrix calculation, eigenvalue decomposition, whitening processing, ICA iteration, and Independent Component (IC) transformation. Timing analysis shows that 99% of the total processing time is consumed by three steps: covariance matrix calculation, whitening processing, and ICA iteration. Therefore, these steps are the main focus of optimization in the study. First, covariance calculation is optimized by load balancing for the lower triangular matrix, mapping the two-dimensional tasks into one-dimensional tasks amenable for parallelization. Second, whitening processing is optimized by interchanging the inner loops to maintain contiguous memory accesses. Similarly, ICA iteration is optimized by maintaining contiguous memory accesses using matrix transpose and temporary array for storage. Compared to the sequential implementation of FastICA, a GPU implementation results in a parallel speedup of 169 times compared to the sequential implementation. When parallelized on the 64-node Tianhe-2 supercomputer, the parallel speedup increases further to reach a value of 410 times.

Kernel Principal Component Analysis (KPCA) is a nonlinear dimensionality reduction algorithm based on the Gaussian kernel and PCA. It generally consists of the following steps: computing Gaussian kernel matrix, performing matrix eigenvalues decomposition, sorting the eigenvalues in descending order, and finally applying KPCA mapping. A study on the performance of the algorithm is conducted in [84] addressing the memory bottleneck issue of a single CPU-GPU heterogeneous node by employing a cluster of such nodes. Three levels of parallelism are presented in the implementation of KPCA, making full use of different platform resources to accommodate large-scale data processing. Instead of coarse-grained parallelization solely based on the Message Passing Interface (MPI), a hybrid implementation utilizing MPI, Open Multi-Processing (OpenMP), and CUDA is utilized to achieve parallel speedup values ranging between 2.75 and 9.27 times. Furthermore, an FPGA implementation of PCA is proposed in [70] and compared to the commercial software version of the algorithm in ENVI software. Benchmarking with the AVIRIS Cuprite image, a speedup of 10 times is obtained when using FPGA-based PCA. Such results make the designed FPGA implementation desirable for onboard data processing of HSI while exhibiting real-time performance with respect to how long it takes the image data to be processed by the targeted hyperspectral device.

#### 4.2.7. HyperLCA

References [49,147,148] are related to the use of the Hyperspectral Lossy Compression Algorithm (HyperLCA). This is a transform-based unmixing-like algorithm designed for high compression ratios. HyperLCA consists of three steps: first, a spectral transform to find the most distinct pixels by means of orthogonal projection techniques is employed. Then, a preprocessing step to prepare the output for entropy encoding is applied. Finally, the entropy encoding is carried out using Golomb-Rice. A hardware-friendly implementation of the algorithm is proposed in [147] using integer arithmetic at different precision levels. The algorithm performance is evaluated on the Xilinx Kintex UltraScaleXQRKU060 FPGA, achieving a throughput of 1.15 MSps with a compression ratio of less than one bit per pixel. Another study [49] implements the transform step on a GPU, and the encoding step is executed as a CPU process. Significant improvement in the throughput is realized, thereby reaching up to 18 MSps by utilizing the algorithm’s high level of parallelism and low computational complexity. For these reasons, HyperLCA is a good compression algorithm candidate for use in systems with tight latency constraints [148], such as onboard satellites. Table 4 shows the summarized details of the collected studies on transform-based compression of remotely sensed hyperspectral images and their related hardware accelerators.

### 4.3. Unmixing-Based Algorithms

**Figure 12:**
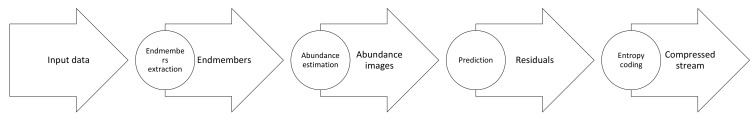
Main steps needed in the unmixing-based compression class of algorithms.

In this section, we discuss research works with hardware accelerations of HSI compression relying on unmixing-based algorithms [38,41,42,43,45,64,86,87]. In this regard, unmixing-based methods broadly consist of two main steps: first, the endmembers are extracted from the hyperspectral image; thus, obtaining spectral signatures that are distinctively different from one another. Second, the abundance of each endmember is calculated for all pixel vectors in the image. This would result in a number of abundance images that are equal to the number of endmembers. The prediction is applied to the resulting abundance images, which are later entropy encoded to obtain the compressed image. Figure 12 shows the overall steps involved in the process of using unmixing-based compression of hyperspectral images.

Compression of hyperspectral images by means of spectral unmixing using the Parallel Pixel Purity Index (P-PPI) algorithm is implemented in various studies [38,41,42,87]. The P-PPI algorithm is applied first to generate a set of endmembers. The fraction of which these endmembers contribute to each pixel vector of the image is estimated using the Parallel Linear Spectral Unmixing (P-LSU) algorithm. Fractional abundance images are then constructed with respect to each endmember. The abundance images are spatially decorrelated using predictive coding before they are eventually passed to the Huffman entropy coder. The proposed solutions are accelerated on the Virtex-II XC2V6000-6 FPGA. They achieved high compression ratios of up to 80:1 while preserving high spectral similarity values to the original image spectra. Such results surpassed those obtained by JPEG2000 and 3D-SPIHT methods. Spectral unmixing is also employed using the GeForce 8800 GTX GPU platform in [43]. In this case, endmember extraction is carried out first using the Pixel Purity Index (PPI) or Automatic Morphological Endmember Extraction (AMEE) algorithms. AMEE is an algorithm based on mathematical morphology operations such as erosion and dilation. These operations are performed by processing the image with a carefully selected set termed Structuring Element (SE). Second, the endmember abundance fractions are estimated using the Fully Constrained Linear Spectral Unmixing (FCLSU) algorithm to devise a lossy compression technique. A high compression ratio of 26:1 is obtained compared to an optimized implementation on a dual-core CPU. The incorporation of DWT into spectral unmixing is evaluated over a heterogeneous network platform in [86]. The idea is to perform a one-dimensional DWT in the spectral direction before broadcasting pixel vectors, skewers, and endmembers by the master node. Results show a reduction in the communication time by 51.4% at the cost of a slight increase in processing time.

Iterative Error Analysis (IEA) is a spectral unmixing algorithm that controls the amount of loss and compression ratio by the number of iterations applied. The calculation of spectral unmixing is performed as more endmembers become available. Because of the lack of dependency within each iteration, the concurrent processing of pixels is possible. Utilizing this important fact, a parallel implementation of the algorithm on Nvidia GeForce GTX 580 GPU is evaluated in [45,64] while maintaining endmembers with acceptable similarity to the reference signatures and yielding a compression ratio of 9.89. Table 5 shows the overall details of the collected studies on unmixing-based compression of remotely sensed hyperspectral images and their hardware accelerations.

### 4.4. Compressive Sensing Algorithms

**Figure 13:**
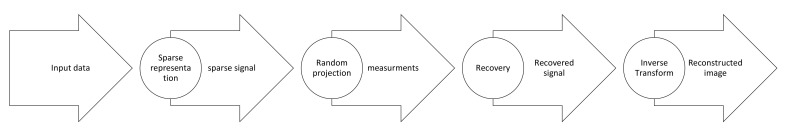
Main steps used in the compressive sensing-based class of HSI compression algorithms.

References [56,57,58,59,61,67,68,69,151] cover Compressive Sensing (CS). In CS, the compressed signal is acquired directly instead of capturing the entire signal. It is an alternative to classical sampling theory as originally postulated by the Nyquist-Shannon sampling theorem. As a result, a small number of incoherent measurements are used to reconstruct the original image [152]. This can reduce the amount of stored and transmitted data and lead to a significant reduction in power requirements. This approach utilizes the sparsity of the image, which is a key property of hyperspectral images. Figure 13 shows the main steps used in compressive sensing-based compression algorithms of HSI.

Hyperspectral Coded Aperture (HYCA) [153] is an algorithm that combines compressive sensing and spectral unmixing. It utilizes the high spectral correlation inherited in hyperspectral data and the fact that the image can be expressed using only a limited number of endmembers. Therefore, the number of measurements needed to reconstruct the image is reduced. The algorithm is first accelerated in [67,68] by evaluating two GPU architectures: GeForce GTX 590 and GeForce GTX TITAN. The study was expanded in [56] to evaluate four different variations of HYCA on the same platform: Parallel HYCA (P-HYCA), P-HYCA-Fast, Parallel Constrained HYCA (P-CHYCA), and P-CHYCA-Fast. In the fast version of P-HYCA, namely, P-HYCA-Fast, the Fast Fourier Transform library cuFF is replaced by a fast iterative method to solve the quadratic problem in the algorithm while delivering a speedup of 1.6 times. In the constrained version of P-HYCA, P-CHYCA, the reconstruction error term is constrained instead of being part of the objective function. A slightly lower speedup is obtained by the constrained versions, P-CHYCA and P-CHYCA-Fast, when compared respectively to P-HYCA and P-HYCA-Fast. The compression ratio is the same for all four variations using AVIRIS Cuprite as the same benchmark image and is equal to 37.6. P-HYCA is also implemented on the Jetson TX1 GPU card in an attempt to reduce the high-power requirements present in the former architectures [57,58]. For the algorithm to operate efficiently on such a low-power platform, the implementation of the algorithm is simplified. First, the integer data type is used instead of the floating-point format. Results show no negative effects of this choice on the accuracy of the reconstructed image. Second, an 8 × 8 window is specified to fit in the shared memory of Jetson TX1, whereas choosing larger window sizes would produce higher execution times. Similar results are obtained using the Jetson TX2 and are presented in [59].

An FPGA acceleration of HYCA is proposed in [61,151], where the algorithm is reorganized to improve data access from the external memory utilizing BIL format. At the architectural level, the system consists of an accelerator and a processor. The processor performs as a controller in addition to data transfer from and to the external memory. The accelerator, on the other hand, runs compute-intensive operations. The proposed solution shows 100 times improvement in power requirement compared to GeForce GTX 590 and GeForce GTX TITAN. It also produces a speedup of 49 times when compared to the Jetson TX2 GPU. On the other hand, the compression ratio is slightly degraded from the GPU-based HYCA. Spectral Compressive Acquisition (SpeCA) is a dimensionality reduction method that suggests a measurement approach based on random projection operating on the spectral domain. The approach relies on the assumption that spectral vectors of real-world hyperspectral images can be well approximated by a Linear Mixing Model (LMM). A parallel implementation of SpeCA is proposed in [69] and shows a speedup of 21 times when compared to its sequential implementation. We provide in Table 6 a detailed summary of the collected studies on compressive sensing-based compression of hyperspectral images and their hardware accelerations.

### 4.5. Vector Quantization-Based Algorithms

**Figure 14:**
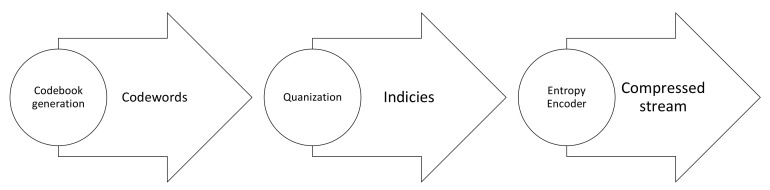
Main steps used in VQ-based class of compression algorithms of HSI.

The VQ-based compression methods exploit the fact that pixels representing the same material have the same spectral information. VQ-based algorithms can obtain higher compression ratios. However, the significant amount of computations required restricts its implementation for real-time applications [31]. VQ-based compression of hyperspectral images consists of two steps: training and coding. First, pixel vectors are grouped based on similarity, and each group is assigned a vector called codevector. A list of all codevectors with their corresponding indices forms a codebook. Second, each pixel vector in the image is replaced by the nearest codevector. An index map of the codevectors is created and transmitted along with the codebook to the decoder for image reconstruction. Figure 14 displays the main steps involved in using VQ-based algorithms for the compression of HSI.

Hyperspectral image compression based on the k-means algorithm is proposed in [37]. It describes a simplified hardware implementation of the algorithm by fixing the precision instead of using floating-point arithmetic in the standard version. In addition, the Euclidian distance metric is replaced by Max and Manhattan metrics to promote a finer degree of parallelism. Higher throughput is obtained, yet it is less optimal than the standard implementation. This is attributed to lower quality clusters when measured by misclassification rate.

Two near-lossless algorithms are developed by the Canadian Space Agency (CSA) based on the VQ method [32]. The first algorithm is called Successive Approximation Multistage Vector Quantization (SAMVQ). As the name suggests, it is built on a multistage structure to reduce computational complexity. The training error is iteratively maintained across all dimensions, and the dimensions that converge faster are excluded from further training. The training and coding are conducted using the Hamming Distance, which is computed faster than the Euclidean Distance. The resulting codebook is much smaller than the conventional VQ-based method, which yields better compression ratios. The second algorithm is called Hierarchical Self-Organizing Cluster Vector Quantization (HSOCVQ). It starts with a small number of codevectors, one for each cluster, and then it proceeds to calculate their fidelity. If the fidelity of a cluster is below a predefined threshold, sub-clusters are generated with their designated codevector. The process iterates until the predefined fidelity is reached for all clusters. The near-lossless feature is evaluated for both SAMVQ and HSOCVQ algorithms at compression ratios of 20:1 and 10:1, respectively. A prototype is accelerated on an FPGA platform with obtained throughput of 614 Mbps, which is about 38 MSps. In Table 7 below, we present the details of the collected studies on VQ-based compression algorithms of remotely sensed hyperspectral images and their hardware accelerations.

### 4.6. Distributed Source Coding-Based Algorithms

**Figure 15:**
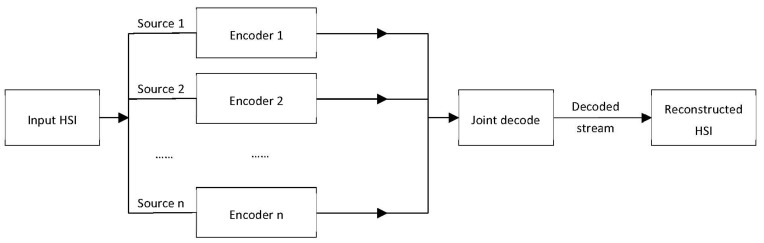
Conceptual framework of distributed source coding-based class of HSI compression algorithms.

Compression based on Distributed Source Coding (DSC) methods shifts the computational complexity from the encoder to the decoder side. Multiple sources provide correlated data yet do not cooperate with one another. Only the decoder side can observe the extra information and jointly decode the received signals. For onboard compression, we are interested in a type of DSC, namely remote source coding, where the received signal presents an extra challenge handling the added noise. DSC-based compression can be of either lossless or lossy type. DSC types and remote source coding are covered in depth in [154]. For hyperspectral data, in particular, one of the advantages of applying DSC is that encoding the current band requires only the correlation information to replicate the predictor; no further computations and buffering are needed [155]. In Figure 15 below, we illustrate the concept of DSC and its use in the compression of HSI.

Prediction-based algorithms utilizing the DSC technique are studied in [100,101]. DSC is accelerated using VHDL on Xilinx Virtex-4 FPGA in [100]. Correlated adjacent bands are compressed independently at the encoder then decoded jointly at the decoder side, where correlation is modeled. DSC shifts the algorithm complexity from the encoder to the decoder, which is convenient for onboard compression where resources are limited. A throughput of 80 MSps is achieved, and compression ratios ranging from 2.4 to 3.5 for raw and calibrated AVIRIS images, respectively, are obtained. DSC is also employed in [101]. Here, the sources are encoded using Low-Density Parity-Check (LDPC) codes and jointly decoded with the joint Bit-Plane Belief Propagation (JBBP) algorithm. Since the decoder side is the most compute-intensive part of the system, it is accelerated using a GPU platform, achieving 20 times in speedup value when compared to the performance of a sequential CPU implementation. Table 8 shows the summarized details of the collected studies on DSC-based compression of remotely sensed hyperspectral images and their hardware accelerations.

### 4.7. Learning-Based Algorithms

**Figure 16:**
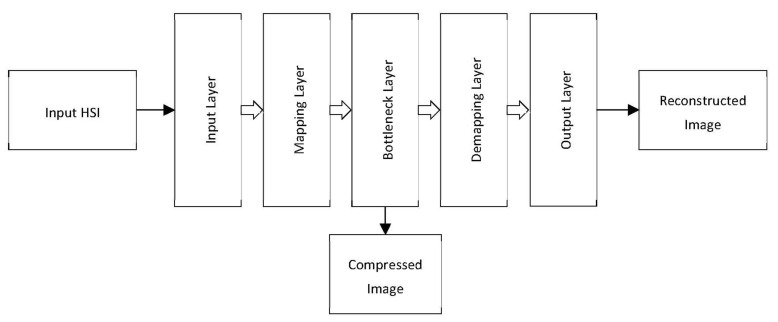
Conceptual framework of hyperspectral image compression and restoration using the learning-based class of algorithms.

The transform-based techniques used for compression, e.g., PCA and wavelet transform, are linear. On the other hand, learning-based techniques such as neural networks make use of probability theory and back-propagation. They allow for solving nonlinear problems, where the compression in the neural network represents the compression of the HSI. Figure 16 shows the general framework of hyperspectral image compression based on the learning method.

Principal Component Analysis is a widely used technique for dimensionality reduction, data compression, and feature extraction. It transforms the highly correlated data into an uncorrelated subspace where the first principal components have the most important features. Nonlinear PCA (NPCA) is implemented by Auto-Associative Neural Networks (AANN), providing better target detection of hyperspectral images at the same compression ratios of the original PCA [39]. The training process is accelerated using a GPU platform. A neural network model is also employed in [83] with a network of nine nodes in the middle layer. The network is trained using 1,440,000 samples accelerated via the use of parallel processing on a GPU.

Deep neural networks are used for nonlinear compression of hyperspectral images in the form of autoencoders (AE) [46]. The employed algorithm is implemented on a cloud computing platform. The performance of AE is compared against PCA using two different activation functions. Similar performance is observed when a linear activation function is used. However, using AEs outperforms PCA when the nonlinear ReLU activation function is utilized instead. Stacked AE is employed in [65] to compress hyperspectral images. A specific soil dataset, namely LUCAS 2009 topsoil, is used to evaluate the impact of the compression system on the prediction of soil properties. The training process is accelerated to roughly 5 min using an Nvidia GTX 970 GPU. Hyperspectral data compression combining spectral clustering and online dictionary learning is proposed in [48]. First, spectral clustering is implemented using graph theory instead of conventional k-means clustering. The method is selected to avoid local optimum, possibly encountered when employing the k-means method. Then, the resulting subclasses from the first phase are used to train the dictionary subclasses. The obtained results show better SNR using the online learning dictionary when compared to transform-based methods at lower compression ratios.

Generative Neural Network (GNN) is employed for hyperspectral image compression in [156], utilizing both spectral and spatial correlations. GNN maps between the latent space, i.e., the bottleneck layer and the image space. Data partitioning is used to increase the compression ratio. However, the use of more blocks yields a lower reconstructed image quality. Weight pruning is also applied to increase the compression ratio with limited loss in the constructed image. It reduces the connections between layers, which implies fewer computations and faster execution. The compression ratio reaches 33:1 with PSNR maintained at around 38 dB, after blocking and pruning. We disclose in Table 9 the details of the collected studies on learning-based compression of remotely sensed hyperspectral images and their hardware accelerations.

## 5. Discussion

In this section, we provide a comparison between the performance of various studies based on the three selected metrics of throughput, power requirement, and Compression Ratio (CR) and according to the used HSI dataset for testing and validation. We then summarize these performance results by ranking the best six hardware-accelerated compression algorithms for HSI using a convenient efficiency metric that combines two of the three mentioned measures related to hardware performance. We also discuss the impact of multiple factors on onboard hyperspectral image compression and their hardware accelerations and provide recommendations for future research. Specifically, we address:

- In Section 5.1, research question RQ3: *What are the comparative performance results, obtained thus far, of the hardware-accelerated HSI compression algorithms*?
- In Section 5.2, Section 5.3, Section 5.4, Section 5.5, research question RQ4: *What are some of the other pertinent factors that can impact the onboard implementation and utilization of hardware-accelerated HSI compression algorithms*?

### 5.1. Performance Comparison

The reviewed studies are grouped by the HSI dataset used to validate the compression system. The goal is to give a meaningful comparison of the system performance according to the following three metrics: compression ratio, throughput, and required power. The majority of the studies evaluated their proposed solution using the AVIRIS dataset. However, to include as many records as possible, the Hyperion imager is also selected for comparison. Records with rare or synthetic hyperspectral images are excluded from the comparison results.

The studies presented in Table 10, Table 11, Table 12, Table 13, Table 14 and Table 15 are grouped based on the following images from HSI datasets: Cuprite, Yellowstone, Hawaii, World Trade Center (WTC), Indian Pines and the Lunar Lake image, respectively. An additional table, Table 16, groups four studies that used hyperspectral images acquired by the Hyperion. Note that the three evaluation metrics used in this review are missing in 24% of the studies and that less than 10% of the studies provide analysis with all these three metrics presented. The highest compression ratio among all studies was reached at 100:1 and is published in [40]. However, this result was re-evaluated by the same two authors in [141] with a focus on obtaining a better image quality where the compression ratio is reduced to 25:1.

The compression ratio of 80:1 is reached using lossy unmixing-based compression algorithms. Their evaluations were achieved using benchmarking with Cuprite, Hawaii and WTC images, respectively (see Table 10, Table 12 and Table 13). Data regarding their throughput and power requirement were not provided. The next-highest compression ratio is reported to be 44.8:1 using compressive sensing, then 40:1 using the transform-based SPIHT algorithm, benchmarked with Cuprite image as shown in Table 10. The corresponding throughput is missing from these studies, as their main focus was on obtaining a high compression ratio. In addition, the highest lossless compression ratio is equal to 3.74. It is produced using a prediction-based algorithm that employs lookup tables. The highest throughput is realized using a prediction-based compression at 219 MSps on an FPGA platform. Similarly, the corresponding compression ratio is missing from this study. Only two studies in Table 10 present full analysis using all three metrics. They are both of lossless type and belong to the prediction-based class of compression algorithms. The best combination of these three metrics is accelerated on a Xilinx Virtex-5 FPGA [93]. This study offers a throughput of 210 MSps while requiring only 0.573 Watts and providing a lossless compression ratio of 2.8.

Studies using benchmarking with the Yellowstone image are presented in Table 11. The highest lossy compression ratio of 8:1 is obtained using a compression algorithm that employs compressive sensing. For lossless compression, the best compression ratios are obtained using the prediction-based clustered DPCM and RLS algorithms at 4.8 and 4.7, respectively [103,104]. Similarly, the remaining two performance metrics are missing from these two studies. The best combination is found in [151] using HYCA compressive sensing with a compression ratio of 8:1, the highest throughput of 391 MSps and low power requirement of 2.6 Watts. It was accelerated on the Xilinx Zynq-7020 SoC platform.

In Table 12, the Hawaii image is utilized in benchmarking and the best lossless compression ratio is obtained at 6.4:1 using the optimized RLS algorithm [104]. The highest throughput is equal to 402.5 MSps generated whilerequiring 11 Watts on a GPU platform employing the CCSDS 123 standard. The best combination of the three metrics is reached via the predictive LCPLC algorithm indicated in [60]. Accelerated on a Xilinx Virtex-7 FPGA, it produced a throughput of 162 MSps with a required power of less than 1 Watt and a compression ratio of 4. However, the adopted scanning order of BSQ might hinder its application for real-time compression.

For studies using the WTC image, they are listed in Table 13. Only one study presents a full analysis using all three metrics [96]. The same study offers the lowest power requirement (0.55 Watts) and a relatively low throughput of 23.3 MSps at a lossless compression ratio of 2.5 while employing the CCSDS 123 standard. Moreover, an FPGA implementation of the same standard produced a much higher throughput of 219.4 MSps while requiring more power (5.30 Watts) [82]. However, no compression ratio was reported in this work. Studies using benchmarking with the Indian Pines image are presented in Table 14. Using a GPU card for acceleration, the highest lossy CR value was reached at 3.2 based on an adaptive predictive LCE algorithm [101]. With a heavy power requirement of 225 Watts, it yields a throughput of 100 MSps. Most of the proposed compression systems in this group that display high throughput values are based on GPUs and are also power hungry. On the other hand, the low-power solutions, which are based on FPGAs, show a reduced throughput not exceeding 11.3 MSps. For all these reasons, the seven studies included in this table are ill-suited for adoption in real-time onboard compression.

Studies presented in Table 15 use the Lunar Lake image as the benchmark for testing. Only a single study disclosed two performance metrics; the rest providing at most one with throughput results missing in all of them. Hence, due to the lack of additional data, it is difficult to decide which work offers the best combination of the three metrics. The last set of studies, presented in Table 16, employ the Hyperion imager as the source of HSI used for evaluation. A high throughput of 750 MSps is obtained in [79] with a power requirement of 0.515 Watts on an FPGA platform. However, no single study within this set has provided a complete analysis using all three metrics. The highest throughput values exhibited in Table 10, Table 11, Table 12, Table 13, Table 14, Table 15 and Table 16 indicate that achieving high throughput is not limited to hardware accelerations using GPUs, but promising results can also be realized on FPGA-based platforms. Similarly, achieving low power requirements is not solely obtained by FPGA-based accelerations of these compression algorithms. In fact, the related results in Table 11 and Table 12 confirm the existence of a few GPU implementations that require less than 11 Watts.

Table 17 shows the highest-ranked six studies based on the highest obtained efficiency value, collected via the synthesized data from the seven tables: Table 10, Table 11, Table 12, Table 13, Table 14, Table 15 and Table 16. Here, efficiency in (MSps/Watts) is calculated by dividing the throughput by the total required power of the compression algorithm. We follow herein the same approach applied in [133] to compare the efficiency of multiple compression implementations. We observe that both the fourth and sixth-ranked algorithms were validated by two sets of HSI: The AVIRIS Yellowstone and Hawaii scenes for the former and the AVIRIS Cuprite and World Trade Center scenes for the latter. Therefore, they appear in Table 11, Table 12, Table 10, and Table 13, respectively. Although this review spans more than 20 years, the most efficient hardware implementations of HSI compression are mostly implemented during the last five years, except for the third most efficient record being published more than ten years ago (2009). Among these six highly ranked hardware accelerations, three were evaluated on SoC platforms while the remaining three were tested using FPGA devices. Moreover, five of the six top-ranked algorithms in terms of efficiency are prediction based while the remaining algorithm from this set is based on compressive sensing.

**Figure 17:**
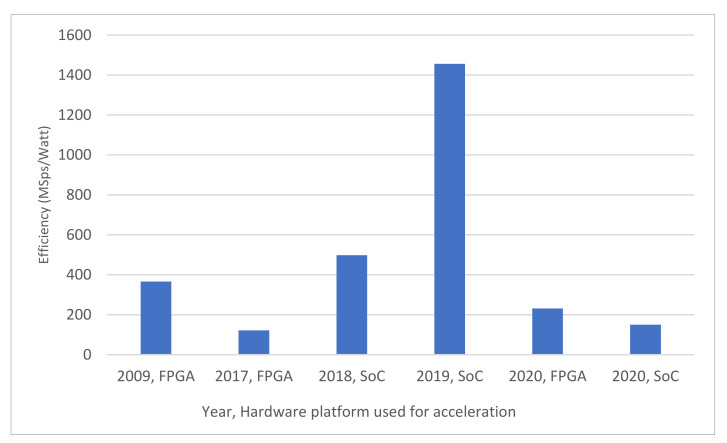
The best efficiency, in MSps/Watt, obtained by six hardware-accelerated implementations of hyperspectral image compression algorithms (the displayed results refer to the works in [49,64,76,115,116,139], respectively).

As also depicted in Figure 17, the most efficient hardware implementation has an efficiency of 1456.0 MSps/Watt. It provides the highest throughput at 750 MSps and requires the second least power at 0.515 Watts. In addition, it uses the BIP format, which is well-suited for real-time compression onboard satellites. Its compression algorithm employs the CCSDS 123 standard and belongs to both the prediction-based class and the lossless compression type. The latter was accelerated on an SoC platform along with the second best compression engine in terms of efficiency. On the other hand, the sixth-ranked compression algorithm among this group provides an efficiency value of 122.1 MSps/Watt, a throughput of 116.0 MSps for sixth best, and a power requirement of 0.95 Watts for fifth best. Likewise, it is a prediction-based and lossless type algorithm that implements the CCSDS 123 compression standard on an FPGA and employs the BIP format. When comparing these two performances in terms of efficiency, we observe that the highest-ranked compression algorithm delivers 11.92 times more efficiency than the sixth best-ranked algorithm. This was obtained as a result of delivering 6.47 times more throughput while also requiring 54.21% less power. Moreover, two of the six listed algorithms are of the lossy compression type. They are ranked fourth and fifth in terms of efficiency while producing compression ratios of 4 and 8, respectively. We expect further improvements in the future since the current most efficient implementations of this compression type have not yet achieved their potential target of higher values of CR. Due to the lack of reported performance data using the three previously mentioned metrics and based on the rankings obtained in Table 17, it is difficult to make further conclusions regarding learning-based, unmixing-based, and transform-based implementations in so far as their efficiency is concerned.

### 5.2. The Impact of Imager Type

There are two types of imagers used in the spatial scanning of HSI: whiskbroom scanners and pushbroom scanners. A whiskbroom scanner uses mirrors that sweep back and forth across the swath to collect data using only few detectors. On the other hand, a pushbroom scanner uses an array of detectors and fewer moving parts. This makes the latter more sensitive to light due to its longer exposure time [157]. However, there exist variations across the detector array of the pushbroom scanner as well. Research efforts in this regard are made to develop better uniformity corrections to reduce these variations [158].

A pushbroom scanner, such as Hyperion and CASI, captures all spectral bands of the scene one line at a time. The long exposure of pushbroom scanners allows for more light to be captured. However, the varying sensitivity of the individual elements of the detector causes the cross-track samples to be different. In whiskbroom scanners, the same element is used to capture cross-track samples. Therefore, more correlations are present when using datasets acquired by whiskbroom scanners than pushbroom, such as AVIRIS and Landsat, which allow for the possibility of higher compression ratios to be attained. For instance, datasets acquired by both whiskbroom and pushbroom scanners are validated in the work presented in [159]. A hyperspectral compression algorithm based on the regression of 3D wavelet coefficients shows compression ratios of 20.85 and 22.12 for pushbroom datasets (Hyperion). The compression ratio employing the same algorithm increased to 26.95 and 27.02 when tested using whiskbroom datasets (AVIRIS). It is noted that this work was excluded from the review because it does not include a hardware-accelerated implementation on one of the previously defined platforms.

### 5.3. The Impact of Scanning Order

The impact of the scanning order on the algorithm dependency is significant since performing a type conversion requires extra memory and additional latency. This is particularly important for real-time applications. Different scan orders have different memory requirements, with BSQ being the most consuming type where the amount of memory required is proportional to the spatial dimension of the sensor. Further, BSQ also shows strong data dependency between adjacent samples, which limits the obtained throughput. The two scanning orders BIL and BIP, require fewer resources in general. However, BIL inherits data dependency between the adjacent samples within a line resulting in a reduced throughput when compared to BIP. The highest throughput is obtained by using the BIP format at one sample per clock cycle. This is difficult to achieve using BIL and BSQ as the inherent data dependency forces the compression engine to take more time and, thus, run slower.

### 5.4. The Impact of Signal-to-Noise Ratio

During radiometric calibration, the image data are corrected according to the sensor’s radiance quality. Calibrated images produce higher compression ratios as they allow the system to make intelligent decisions. Raw pushbroom data exhibit artifacts that hinder the compressor from reaching its full potential, unlike calibrated data or images acquired by whiskbroom scanners. For instance, hyperspectral data acquired by Walsh Hadamard spectrometer have high SNR and thus are expected to produce high compression ratios [54].

### 5.5. Power Considerations

In order to investigate the power considerations for the compression system, we assume the use of CubeSats as the host for such a system. CubeSats, known for their modularity, are categorized by a standard size and weight: the one-unit CubeSat (1U) is 10 × 10 × 10 cm, the two-unit CubeSat (2U) is 10 × 10 × 20 cm, and the three-unit CubeSat (3U) is 10 × 10 × 30 cm. The largest weight is 1 kg per unit size, e.g., 3 kg for a 3U CubeSat. This limited size restricts the area available for solar panels [9]. Based on the NanoSat Database [160], the smallest launched CubeSat is 0.25U, and the largest is 12U, which took place in January 2019. The launch of 16U and 20U CubeSats are expected to take place in the near future.

According to AAC Clyde Space [161], a company specializing in CubeSat manufacturing, the peak payload power for 1U, 3U, 6U and 12U CubeSats are 15, 60, 120, and 240 Watts, respectively. The typical FPGA power requirements range between 5 and 10 Watts for Virtex and Stratix FPGAs. Devices requiring less power are also available, such as Xilinx Spartan, Altera Cyclone, and Xilinx Artix families, with power requirements ranging from 1 to 2.5 Watts [9]. Among the SoC platforms covered in this review, the largest required power was reached by Xilinx Zynq Z7045Q SoC at 9 Watts [109]. Comparing this to the GPU power requirements, it is reported that, for instance, the Nvidia GPUs require power that ranges between 75 and 365 Watts [162]. It follows that the number of CubeSat units needed when using GPUs will have to increase due to the limited power budget of the former. This would lead to further increases in the cost of acquiring and launching such satellite systems. It is especially true when knowing that the estimated cost to build one CubeSat unit is between 50,000 and 200,000 USD and that a similar amount is required for the launching of the built system [163].

Based on the above analysis, FPGA-based platforms provide a clear advantage when compared to GPUs in terms of power requirements except for the Jetson GPU accelerators, as theirs range between 5 and 30 Watts. However, the lack of radiation-hardened GPUs imposes enabling of the hardening via software and the use of extra memory for data integrity, which further exacerbates the amount of power required by the system. Nonetheless, GPUs show higher flexibility and lower development time. In terms of throughput, the Jetson accelerators present a good tradeoff. On the other hand, all the GPU-accelerated studies in this review validated their implementations using only the BSQ format. This format is well-suited for real-time compression for snapshot imagers since the spatial data across all bands are captured simultaneously. However, we found no relevant studies that employ GPUs with either the BIL or BIP scanning formats. If the latter were true, it could be also convenient for pushbroom and whiskbroom scanners to achieve real-time compression. By real-time we mean performing the compression upon data arrival during the acquisition process. According to [164], this is also called online compression, as opposed to the offline approach where compression is started after data have been stored. Real-time compression allows for more data to be captured before it gets overwritten due to limited storage capacity. This is particularly important for massive data sizes such as those obtained by hyperspectral imagers and sounders.

### 5.6. Current Research Gaps

Over the course of this review, we observed that only few studies investigated the feasibility of learning-based techniques for onboard compression of HSI. They are mostly lossy algorithms and are mainly implemented using GPU architectures. So far, no studies were found using FPGA-based platforms for the acceleration of learning algorithms designed for compression of remotely sensed hyperspectral images.

The strength of compressive sensing, besides the small number of measurements, is that the technique is blind and does not require prior knowledge of the image characteristics. Although CS is intriguing, it appears that all the available hardware-accelerated implementations so far are attempted by one group of researchers [56,57,58,59,61,67,68,69,151]. In addition, the FPGA-accelerated compression algorithms by means of unmixing have been implemented using only high-level synthesis tools. We believe that more efficient implementations could be accomplished by considering direct hardware implementations using HDLs.

Prediction-based techniques have proved their suitability for onboard compression due to their simplicity and low memory requirements compared to other techniques such as transform-based and dictionary-based methods. The error propagation problem of this technique could be constrained by applying block-based compression. While there are many parallel implementations of the CCSDS standard in the literature using both GPU and FPGA-based architectures, other prediction-based techniques remain overlooked thus far. The latter could become, after some adaptation, valuable future candidates to enhance the current state of the art in HSI compression. Transform-based compression algorithms provide higher compression ratios with lower throughput due to the high amount of computations needed. We note that most of the transformed-based studies focus their optimization on only one of these metrics. In some of these studies, there were limited results and a lack of information concerning other metrics. Consequently, this could complicate the decision-making process of how to proceed forward with improving some of these compression algorithms.

### 5.7. Future Recommendations

Given the current state of hardware-accelerated compression of remotely sensed hyperspectral images, we make the following suggestions for future research:

- More research work needs to be focused on hardware-accelerated compression by means of learning-based and compressive-sensing techniques in order to enrich the state of the art in this area.
- The full potential of hardware-accelerated compression using unmixing algorithms is not fully explored. Unmixing techniques can be further simplified to reduce their complexity. The power of this technique is manifest in the provision of both compression and classification, which is the purpose of obtaining spectral signatures in the first place.
- As space agencies around the world make available a variety of hyperspectral data for the research community, different datasets should be considered in the same study to present results that are unbiased by calibration or scanner type.
- Researchers are encouraged to provide more information regarding the performance of the implemented compression algorithm in terms of a full range of metrics such as compression ratio, throughput, and power requirement. This is in addition to SNR in order to better support decision making in regards to the best tradeoffs needed for further improvements.
- Explore other transform-based techniques for compression of HSI as the current methods are mainly focused on three transforms: DWT, DCT, and KLT.
- The use of Synthetic Radar Aperture (SAR) data types for hyperspectral image compression should be studied further. These data types might be promising in terms of obtaining more efficient compression because the coherence data from SAR images could be employed to detect different levels of changes in the scene. This is due to the fact that SAR’s performance is independent of visibility and available daylight.
- The use of Machine Learning (ML) techniques and models to solve many engineering and scientific problems is increasing at a rapid pace as ML is becoming less domain-specific and more general purpose than ever before [165]. To deliver on the high potential of ML, the design of domain-specific architectures tailored specifically for machine learning is paramount in this regard [166]. Given that ML has become a powerful prediction tool for the analysis and processing of hyperspectral data [167], we recommend exploring these new hardware platforms for the acceleration of HSI compression.

## 6. Conclusions

We present in this paper a systematic review study of hardware-accelerated compression algorithms for remotely sensed hyperspectral images spanning more than 21 years of research works published in recognized journals and conferences. In order to include the research papers that would facilitate answering the research questions, a careful selection strategy has been followed using the PRISMA protocol. We also provide a comparative analysis of the collected research papers to glean the emerging hardware architectures most suitable for HSI compression according to the dataset used and based on suitable evaluation metrics that include compression ratio, throughput, power requirement, and efficiency. The best compression ratios are generally obtained by unmixing-based algorithms, while prediction-based methods produce faster results in terms of higher efficiency and throughput. Further, power requirement is mainly characterized by the underlying computing platform, whereas the choice of hardware architecture is driven by the nature of the compression algorithm. Due to the high number of computations involved, the unmixing-based and the transform-based algorithms are the most computationally demanding methods. In terms of hardware accelerators, FPGAs, GPUs, and SoCs will continue to be the most adopted platforms for HSI compression, especially as future improvements in their clock speed, throughput, memory capacity, and power requirement are attained.

The review shows the rapid increase of research in this area over the last 11 years, about 3.6 times the number of publications compared to the first half. In particular, the studies published from 2012 onwards are dominated by the CCSDS standard, potentially driving the research trend away from the other compression techniques. As expected, the data obtained by the AVIRIS imager are the most widely used benchmark, found in about 76.24% of the studies. Although AVIRIS is a high-resolution whiskbroom imager, it is less likely to be used for small satellites such as CubeSats since the mechanics of the moving parts make such scanners expensive in terms of power requirements and development cost.

We conclude by stating that the full potential of hardware-accelerated compression techniques has not been fully realized yet. This can be inferred by the myriad of compression algorithms solely covered in software-based reviews over the last decade. Nonetheless, these recent works have enriched the field with many learning-based techniques. We recommend that researchers consider such HSI compression algorithms when designing for high-performance solutions as these methods could be excellent candidates for hardware accelerations on different platforms.

## Figures and Tables

**Table 1:** The areas of interest covered by related reviews on HSI image compression algorithms.

Authors, Year	Area of Interest
(Lambert-Nebout and Moury, 1999)	Lossy compression algorithms used onboard CNES missions.
(Yu, Vladimirova et al., 2009)	Lossless and lossy image compression systems onboard space missions with a focus on multispectral images.
(Mat Noor and Vladimirova, 2013)	Lossless compression techniques of HSI onboard spaceborne hyperspectral missions.
(Lopez, Vladimirova et al., 2013)	FPGA-based HSI compression and linear unmixing techniques in remote sensing applications.
(Sanjith and Ganesan, 2014)	A short review on HSI compression algorithms covering lossless, lossy, and near-lossless algorithms.
(Babu, Ramachandran et al., 2015)	Statistical and wavelet-based compression algorithms of hyperspectral images.
(Rusyn, Lutsyk et al., 2016)	FPGA-based lossless image compression techniques in remote sensing applications.
(Dusselaar and Paul, 2017)	Intra-band and inter-band compression of hyperspectral images at the algorithm level.
(Gunasheela and Prasantha, 2018)	Multispectral and hyperspectral image compression algorithms onboard satellites.
(Hussain, Al-Fayadh et al., 2018)	Lossless and lossy compression algorithms with the focus on medical images.
(Dua, Kumar et al., 2020)	Classification of HSI compression algorithms according to multiple parameters.

**Table 2:** List of studies excluded in this review using our search criteria.

Excluded Studies	Reason for Exclusion
[71]	The paper addresses only the image reconstruction phase of compressive sensing.
[72]	The described application is not related to remote sensing (medical imaging).
[73]	The algorithm is intended for large video data, e.g., high-definition television (HDTV), and is not validated using hyperspectral data.
[74]	The compression technique is validated using a gray-scale image.
[75]	The compression algorithm is not accelerated using one of the defined hardware platforms.
[76]	The compression algorithm is not accelerated using one of the defined hardware platforms.
[77]	The paper was published after the cutoff deadline required for inclusion in this review.

**Table 3:** List of studies on prediction-based compression systems of hyperspectral images and their hardware platforms.

Compression Algorithm	Hardware Platform	Programming Method	Ref.
Fast Lossless (adaptive filtering-Rice code)	Xilinx Virtex-IV LX160 FPGA	-	[103]
VS-3DGAP-ExtRice (CCSDS based)	Xilinx Virtex-5 FPGA	Matlab-AccelDSP	[93]
Fast Lossless (adaptive filtering-Rice code)	Xilinx Virtex-IV LX25 FPGA	-	[104]
LP-CC	Nvidia GeForce 9600 GT GPU	CUDA	[122]
FELICS based- Improved Prediction-Simplified Rice	FPGA-embedded CPU	-	[110]
Median prediction-LUTs-Adaptive Arithmetic Coding	Xilinx Spartan3 XC3S4000 FPGA-ARM926EJ-S processor	-	[114]
(MED-GAP)-Huffman Coding	Xilinx SPARTAN-3E FPGA	Verilog	[95]
FELICS based- Improved Prediction-Simplified Rice	Radiation tolerant FPGA	-	[111]
LCE	Nvidia Tesla C2075 GPU	CUDA	[116]
Fast Lossless-Adaptive Linear Prediction	Nvidia GeForce GTX 580 GPU	CUDA	[105]
CCSDS Lossless	Nvidia GeForce 560M GTX GPU	OpenMP	[123]
RHBSW (FL-based)	Xilinx Virtex-4 FX60 FPGA ×2	-	[107]
LCE	Nvidia Tesla C2075-GeForce GTX 480 GPUs	CUDA	[55]
RHBSW (FL-based)	Xilinx Virtex-4 FX60 FPGA	-	[108]
Adaptive predictive-LCE	Xilinx Virtex-5 5VFX130 FPGA	CatapultC HLS tool	[88]
Adaptive predictive-LCE	Xilinx Virtex-5 FPGA	CatapultC HLS tool	[89]
Adaptive predictive-LCE	Nvidia Tesla C2075 GPU	CUDA	[117]
Fast Lossless-Adaptive Linear Prediction	Xilinx Virtex-V-VI FPGA	Verilog	[106]
LCE	Microsemi RTAX2000 FPGA	CatapultC HLS tool	[90]
Inter-band and Intra-band Prediction based	Xilinx Virtex-5 Pro FPGA	Verilog	[115]
LCE	Xilinx Virtex-5 FPGA/Actel RTAX2000S	CatapultC HLS tool	[91]
Extended CCSDS 123	Xilinx Virtex-5 XQR5VFX130 FPGA	-	[124]
CCSDS-123.0-B-1	Xilinx Virtex-5QV FX130T FPGA	-	[125]
C-DPCM	Nvidia Tesla K20C GPU	CUDA	[140]
CCSDS 123 based (HyLoc)	Microsemi RTAX- Xilinx Virtex-4,5 FPGAs	VHDL	[80]
SHyLoc (CCSDS 123 and 121)	FPGA ^1^	SystemC	[10]
CCSDS123	Nvidia GeForce GTX 750 Ti GPU	CUDA	[126]
CCSDS 123	Xilinx Virtex-4,5,7 FPGAs-Nvidia GT 440, 610 GPUs	VHDL	[82]
Optimized RLS	Nvidia Kepler GTX 690 GPU	CUDA	[120]
CCSDS 123	Xilinx Virtex-4,7 FPGA	VHDL	[96]
CCSDS123	SoC FPGA ARM Cortex-A9 MPCore	-	[136]
CCSDS 123	Xilinx Zynq-7020 SoC	VHDL	[134]
CCSDS 123	Nvidia GeForce GTX 750 Ti GPU-Jetson TX1 board	CUDA	[127]
CCSDS 123	Xilinx Zynq-7000 SoC	Vivado HLS-Catapult C	[92]
FLEX	Xilinx Zynq Z7045Q SoC	-	[109]
CCSDS 123	Xilinx Zynq-7000 SoC	VHDL	[128]
CCSDS 123	Xilinx Zynq-7035 SoC	VHDL	[79]
CCSDS 123	Xilinx Zynq-7000 SoC	VHDL	[130]
Clustered DPCM-Prediction based	Nvidia GeForce GTX 1080Ti-TITAN X GPU	CUDA	[119]
CCSDS 123	Nvidia Jetson TX2 board	CUDA	[131]
LCPLC	Xilinx Virtex-7 FPGA	VHDL and Verilog	[60]
CCSDS 123	Xilinx Zynq-7045 SoC	HDL	[135]
SHyLoc (CCSDS 123-CCSDS 121)	Xilinx Virtex-5 FX 130T FPGA, Microsemi RTG4	VHDL	[97]
CCSDS 123-bit rate control stage	Xilinx Zynq UltraScale+ FPGA-based MPSoC	VHDL	[50]
SHyLoc	Xilinx Virtex XQR5VFX130 FPGA	VHDL	[98]
CCSDS	Xilinx Kintex-7 FPGA	Verilog	[139]
CCSDS123	Xilinx Zynq-7000 SoC	Verilog	[137]
SHyLoc	Xilinx Zynq-7000 SoC	VHDL	[99]
CCSDS123	Nvidia Jetson (Nano-TX2-Xavier) GPU	CUDA	[133]
CCSDS123	Xilinx Kintex-7 FPGA	-	[138]
RLS	PARAM-SHIVAY supercomputer	Python	[85]
CCSDS123	Nvidia Jetson TX2 board	CUDA	[132]
CCSDS 123	Xilinx Virtex-5QV FPGA	VHDL	[81]

^1^ Implemented on FPGA platform, no specific hardware model is mentioned.

**Table 4:** List of studies on transform-based compression systems of hyperspectral images and their hardware platforms.

Compression Algorithm	Hardware Platform	Programming Method	Ref.
Fixed-Order SPIHT	Xilinx Virtex 2000E FPGAs ×3	VHDL	[52]
Anomaly detection-wavelet-based transform	DSP-Xilinx XCV1000-XCV300 FPGAs	-	[40]
Anomaly detection-JPEG2000	DSP-Xilinx XCV1000-XCV300 FPGAs	-	[141]
Linear prediction-SPIHT	(FPGA ^1^) ×2	-	[63]
Wavelet-JPEG2000	Xilinx Virtex-4-Xilinx Virtex-II FPGAs	-	[53]
JPEG2000	Xilinx FPGA-ADV212	-	[54]
MPEG-4	H.264/AVC encoder	-	[44]
JPEG2000 (Integer DWT, No quantization)	Nvidia GeForce GTX 480 GPU	CUDA	[47]
Integer KLT	Actel SoC (ARM Cortex M-3-flash-based FPGA)	-	[144]
KLT-JPEG2000	Nvidia GeForce GTX580 GPU	CUDA	[149]
JPEG-LS	Nvidia GTX480 GPU	CUDA	[143]
KLT	Intel Cyclone IV FPGA-ARM Cortex M-3 Processor	HDL	[78]
KLT-JPEG2000	Nvidia GeForce GTX580 GPU	CUDA	[150]
POT	Microsemi RTAX2000S-DSP FPGA	-	[145]
FastICA	(8-core Intel Xeon E5-2650 CPUs-Nvidia Tesla K20c GPUs) ×2	CUDA/OpenMPI	[146]
K-means-PCA-DWT-USDZQ-AC	Nvidia GeForce GTX 750 Ti GPU	CUDA	[66]
KPCA	Nvidia Tesla K20c GPU ×2-Intel Xeon CPU E5-2670 GPU	CUDA	[84]
PCA	Xilinx Virtex-7 XC7VFX690T FPGA	VHDL	[70]
HyperLCA	Nvidia Jetson TK1 GPU	CUDA	[49]
JYPEC (PCA-JPEG2000)	Xilinx Virtex-7 XC7VX690T FPGA	VHDL	[142]
HyperLCA	Xilinx Zynq-7000 SoC	VHDL-HLS	[51]

^1^ FPGA platform is used, but device model is unspecified.

**Table 5:** List of studies on unmixing-based compression systems of hyperspectral images and their hardware platforms.

Compression Algorithm	Hardware Platform	Programming Method	Ref.
P-PPI-P-LSU	Xilinx Virtex-II XC2V6000-6 FPGA	Handel-C	[41]
P-PPI-P-LSU	Xilinx Virtex-II XC2V6000-6 FPGA	Handel-C	[38]
PPI-LSU	Xilinx Virtex-II XC2V6000-6 FPGA	Handel-C	[42]
PPI-Spectral Unmixing	Xilinx Virtex-II XC2V6000-6 FPGA	Handel-C	[87]
PPI or AMEE-FCLSU	Nvidia GeForce 8800 GTX GPU	CUDA	[43]
DWT-Spectral Unmixing	(Heterogeneous Workstations) ×16	C++	[86]
IEA	Nvidia GeForce GTX 580 GPU	CUDA	[64]
IEA	Nvidia GeForce GTX 580 GPU	CUDA	[45]

**Table 6:** List of studies on compressive sensing-based compression systems of hyperspectral images and their hardware platforms.

Compression Algorithm	Hardware Platform	Programming Method	Ref.
P-HYCA	Nvidia GeForce GTX 590-GeForce GTX TITAN GPUs	CUDA	[67]
P-HYCA	Nvidia GeForce GTX 590-GeForce GTX TITAN GPUs	CUDA	[68]
P-HYCA-P-HYCA-FAST-P-CHYCA-P-CHYCA-FAST	Nvidia GeForce GTX 590-GeForce GTX TITAN GPUs	CUDA	[56]
SpeCA	Intel i7-4790 CPU-Nvidia GeForce GTX 980 GPU	CUDA	[69]
P-HYCA	Nvidia Jetson TX1 GPU board	CUDA	[57]
P-HYCA	Nvidia Jetson TX1 GPU board	CUDA	[58]
P-HYCA	Nvidia Jetson TX2 GPU board	CUDA	[59]
HYCA	Xilinx Zynq-7020 SoC	VHDL	[151]
HYCA	Zynq Zedboard with a XC7Z020 SoC FPGA	VHDL	[61]

**Table 7:** List of studies on VQ-based compression systems of hyperspectral images and their hardware platforms.

Compression Algorithm	Hardware Platform	Programming Method	Ref.
K-means clustering	FPGA ^1^	-	[37]
SAMVQ and HSOCVQ	Xilinx Virtex-II FPGA	-	[32]

^1^ Implemented on FPGA, but no specific hardware model is mentioned.

**Table 8:** List of studies on DSC-based compression systems of hyperspectral images and their hardware platforms.

Compression Algorithm	Hardware Platform	Programming Method	Ref.
Scaler Coset Codes	Xilinx Virtex-4 FPGA	VHDL	[100]
LDPC-JBBP	Nvidia GTX480 GPU	-	[101]

**Table 9:** List of studies on learning-based compression algorithms of hyperspectral images and their hardware platforms.

Compression Algorithm	Hardware Platform	Programming Method	Ref.
AANN-NLPCA	Intel Sandy Bridge GPU	-	[39]
Graph theory-based clustering-Online learning dictionary	Nvidia GPU ^1^	-	[48]
Neural Network	Nvidia GeForce GTX 650 Ti GPU-Intel Core-i7-870 CPU	Python	[83]
Autoencoder	JetStream Cloud Services	Openstack	[46]
GNN	Nvidia GTX 1060 GPU	-	[156]
Autoencoder	Nvidia GTX 970 GPU	-	[65]

^1^ GPU model is unspecified.

**Table 10:** Comparison of the proposed compression system validated using the AVIRIS Cuprite image.

Compression Algorithm	Algorithm Class	CR	Throughput (MSps)	Power (Watts)	Efficiency (MSps/W)	Hardware Platform	Programming Method	Scanning Order	Ref.
Linear prediction-SPIHT	Transform-based (Lossy)	40	-	-	-	(FPGA) ×2	-	-	[63]
P-PPI-P-LSU	Unmixing-based (Lossy)	80	-	-	-	Xilinx Virtex-II XC2V6000-6 FPGA	Handel-C	-	[41]
SAMVQ and HSOCVQ	VQ-based (Near-Lossless)	20	38	-	-	Xilinx Vertext-II FPGA	-	-	[32]
P-PPI-P-LSU	Unmixing-based (Lossy)		-	-	-	Xilinx Virtex-II XC2V6000-6 FPGA	Handel-C	-	[38]
PPI-LSU	Unmixing-based (Lossy)	80	-	-	-	Xilinx Virtex-II XC2V6000-6 FPGA	Handel-C	-	[42]
Scaler Coset Codes	DSC-based (Lossless)	2.9	80	-	-	Xilinx Virtex-4 FPGA	VHDL	BSQ	[100]
VS-3DGAP-ExtRice (CCSDS based)	Prediction-based (Lossless)	2.8	210	0.573	366.5	Xilinx Virtex-5 FPGA	Matlab-AccelDSP	-	[93]
Median prediction-LUTs-Adaptive Arithmetic Coding	Prediction-based (Lossless)	3.74	16.5	-	-	Xilinx Spartan3 XC3S4000 FPGA-ARM926EJ-S processor	-	-	[114]
MPEG-4	Transform-based (Lossy)	16	-	-	-	H.264/AVC encoder	-	-	[44]
FELICS based-Improved Prediction-Simplified Rice	Prediction-based (Lossless)	1.7–2.7	30	-	-	Radiation tolerant FPGA	-	-	[111]
JPEG2000 (Integer DWT, No quantization), JPEG2000	Transform-based (Lossless, Lossy)	2–13	-	250	-	Nvidia GeForce GTX 480 GPU	CUDA	-	[47]
Integer KLT	Transform-based (Lossless)	-	-	0.25	-	Actel SoC (ARM Cortex M-3 microcontroller-flash-based FPGA)	-	-	[144]
IEA–Unmixing	Unmixing-based (Lossy)	9.89	-	244	-	Nvidia GeForce GTX 580 GPU	CUDA	-	[45]
KLT-JPEG2000	Transform-based (Lossy)	-	-	224	-	Nvidia GeForce GTX580 GPU	CUDA	-	[149]
JPEG-LS	Transform-based (Lossless)	2.2	-	250	-	Nvidia GTX480 GPU	CUDA	-	[143]
IEA	Unmixing-based (Lossy)	9.89	-	244	-	Nvidia GeForce GTX 580 GPU	CUDA	-	[64]
KLT-Integer KLT	Transform-based (Lossless, Lossy)	-	-	2–1.3	-	Intel Cyclone IV FPGA, ARM Cortex M-3 Processor	HDL	-	[78]
KLT-JPEG2000	Transform-based (Lossy)	-	-	224	-	Nvidia GeForce GTX580 GPU	CUDA	-	[150]
P-HYCA	CS-based (Lossy)	37.6	-	365–250	-	Nvidia GeForce GTX 590-GeForce GTX TITAN GPUs	CUDA	-	[67]
P-HYCA	CS-based (Lossy)	37.6–14.93	-	365–250	-	Nvidia GeForce GTX 590-GeForce GTX TITAN GPUs	CUDA	-	[68]
P-HYCA-P-HYCA-FAST-P-CHYCA-P-CHYCA-FAST	CS-based (Lossy)	44.8–14.93–37.6	-	365–250	-	Nvidia GeForce GTX 590-GeForce GTX TITAN GPUs	CUDA	-	[56]
Prediction-based	CCSDS 123 (Lossless)	-	179.7	3.04	59.1	Xilinx V-5QV FX130T FPGA	VHDL	BIP	[82]
Prediction-based	CCSDS 123 (Lossless)	-	116.0	0.95	122.1	Xilinx V-4 XC2VFX60 FPGA	VHDL	BIP	[82]
Prediction-based	CCSDS 123 (Lossless)	-	219.4	5.30	41.4	Xilinx V-7 XC7VX690T FPGA	VHDL	BIP	[82]
Prediction-based	CCSDS 123 (Lossless)	-	62.2	65	0.96	Nvidia GT 440 GPU	OpenCL	BIP	[82]
Prediction-based	CCSDS 123 (Lossless)	-	62.6	29	2.2	Nvidia GT 610 GPU	OpenCL	BIP	[82]
Graph theory-based clustering-Online learning dictionary	Learning-based (Lossy)	5.3	-	-	-	Nvidia GPU	-	-	[48]
Prediction-based	CCSDS 123 (Lossless)	2.5	23.3	0.55	42.4	Xilinx Virtex-4 FPGA	VHDL	All	[96]
Prediction-based	CCSDS 123 (Lossless)	2.5	47.6	-	-	Xilinx Virtex-7 FPGA	VHDL	All	[96]
PCA	Transform-based (Lossy)	-	-	-	-	Xilinx Virtex-7 XC7VFX690T FPGA	VHDL	-	[70]
JYPEC (PCA-JPEG2000)	Transform-based (Lossy)	-	23.75	-	-	Xilinx Virtex-7 XC7VX690T FPGA	VHDL	-	[142]
HYCA	CS-based (Lossy)	-	-	3.66	-	Zynq Zedboard with a XC7Z020 SoC FPGA	VHDL	BIL	[61]

**Table 11:** Comparison of the proposed compression system validated using the AVIRIS Yellowstone image.

Compression Algorithm	Algorithm Class	CR	Throughput (MSps)	Power (Watts)	Efficiency (MSps/W)	Hardware Platform	Programming Method	Scanning Order	Ref.
CCSDS123	Prediction-based (Lossless)	3.4	3.5	0.169	20.7	Microsemi RTAX FPGA	VHDL	BSQ	[80]
CCSDS123	Prediction-based (Lossless)	3.4	11.3	2.345	4.8	Xilinx Virtex-4 FPGA	VHDL	BSQ	[80]
CCSDS123	Prediction-based (Lossless)	3.4	11.2	2.345	4.8	Xilinx Virtex-5 FPGA	VHDL	BSQ	[80]
Optimized RLS	Prediction-based (Lossless)	4.7	-	-	-	Nvidia Kepler GTX 690 GPU	CUDA	-	[120]
CCSDS123	Prediction-based (Lossless)	-	20.4	-	-	Xilinx Zynq-7000 SoC	VHDL	BIP	[128]
CCSDS123	Prediction-based (Lossless)	3.2–4	165.65	2.6	63.7	Xilinx Zynq-7000 SoC	VHDL	BSQ	[130]
Clustered DPCM-Prediction based	Prediction-based (Lossless)	4.8	280	650	0.4	Nvidia TITAN X GPU	CUDA	-	[119]
CCSDS 123	Prediction-based (Lossless)	1.5–5.5	-	-	-	Nvidia Jetson TX2 board	CUDA	-	[131]
LCPLC	Prediction-based (Lossy)	4.3	162	0.7	231.4	Xilinx Virtex-7 FPGA	VHDL and Verilog	BSQ	[60]
LCPLC	Prediction-based (Lossy)	4.3	119.96	2.73	43.9	Xilinx Virtex-5 FPGA	VHDL and Verilog	BSQ	[60]
HYCA	CS-based (Lossy)	8	391	2.6	150.4	Xilinx Zynq-7020 SoC	VHDL	BIL	[151]
CCSDS123	Prediction-based (Lossless)	-	45	5.7	7.9	Nvidia GPU Jetson (Nano)	CUDA	BSQ	[133]
CCSDS123	Prediction-based (Lossless)	-	146.9	6.28	23.4	Nvidia GPU Jetson (TX2)	CUDA	BSQ	[133]
CCSDS123	Prediction-based (Lossless)	-	308.13	10.9	28.3	Nvidia GPU Jetson (Xavier)	CUDA	BSQ	[133]
RLS	Prediction-based (Lossless)	-	-	-	-	PARAM-SHIVAY supercomputer	Python	BIL	[85]
CCSDS123	Prediction-based (Lossless)	-	69.8	4.56	15.3	Nvidia Jetson TX2 board	CUDA	-	[132]

**Table 12:** Comparison of the proposed compression system validated using the AVIRIS Hawaii scene.

Compression Algorithm	Algorithm Class	CR	Throughput (MSps)	Power (Watts)	Efficiency (MSps/W)	Hardware Platform	Programming Method	Scanning Order	Ref.
Linear prediction-SPIHT	Transform-based (Lossy)	40	-	-	-	(FPGA) ×2	-	-	[63]
P-PPI-P-LSU-Predictive coding spatially-Huffman	Unmixing-based (Lossy)	80	-	-	-	Xilinx Virtex-II XC2V6000-6 FPGA	Handel-C	-	[41]
RHBSW (FL-based)	Prediction-based (Lossless)	-	2.58	-	-	Xilinx Virtex-4 FX60 FPGA ×2	-	-	[107]
Inter-band and Intra-band Prediction based	Prediction-based (Lossless)	3.28	-	1.194	-	Xilinx Virtex-5 Pro FPGA	Verilog	BIP	[115]
CCSDS123	Prediction-based (Lossless)	2.2–4.5	183.4	60	3.1	Nvidia GeForce GTX 750 Ti GPU	CUDA	-	[126]
Optimized RLS	Prediction-based (Lossless)	6.4	-	-	-	Nvidia Kepler GTX 690 GPU	CUDA	-	[120]
CCSDS 123	Prediction-based (Lossless)	-	116–401	15–60	6.7–7.7	Nvidia GTX 750 Ti GPU-Jetson TX1 board	CUDA	-	[127]
FLEX	Prediction-based (Lossless)	-	-	9	-	Xilinx Zynq Z7045Q SoC	-	-	[109]
Clustered DPCM-Prediction based	Prediction-based (Lossless)	5	233	-	-	Nvidia TITAN X GPU	CUDA	-	[119]
CCSDS 123	Prediction-based (Lossless)	1.5–5.5	129	4.9	26.3	Nvidia Jetson TX2 board	CUDA	-	[131]
LCPLC	Prediction-based (Lossy)	4	162	0.7	231.4	Xilinx Virtex-7 FPGA	VHDL and Verilog	BSQ	[60]
LCPLC	Prediction-based (Lossy)	4	119.96	2.73	43.9	Xilinx Virtex-5 FPGA	VHDL and Verilog	BSQ	[60]
CCSDS123	Prediction-based (Lossless)	-	66	5.7	11.6	Nvidia GPU Jetson (Nano)	CUDA	BSQ	[133]
CCSDS123	Prediction-based (Lossless)	-	203.3	6.28	32.3	Nvidia GPU Jetson (TX2)	CUDA	BSQ	[133]
CCSDS123	Prediction-based (Lossless)	-	402.5	10.9	36.9	Nvidia GPU Jetson (Xavier)	CUDA	BSQ	[133]
RLS	Prediction-based (Lossless)	-	-	-	-	PARAM-SHIVAY supercomputer	Python	BIL	[85]
CCSDS123	Prediction-based (Lossless)	-	93.2	4.56	20.4	Nvidia Jetson TX2 board	CUDA	-	[132]

**Table 13:** Comparison of the proposed compression system validated using the AVIRIS World Trade Center image.

Compression Algorithm	Algorithm Class	CR	Throughput (MSps)	Power (Watts)	Efficiency (MSps/W)	Hardware Platform	Programming Method	Scanning Order	Ref.
PPI Spectral Unmixing	Unmixing-based (Lossy)	80	-	-	-	Xilinx Virtex-II XC2V6000-6 FPGA	Handel-C	-	[87]
PPI or AMEE-FCLSU	Unmixing-based (Lossy)	80	-	-	-	Nvidia GeForce 8800 GTX GPU	CUDA	-	[43]
Daubechies wavelet	Unmixing-based (Lossy)	-	-	-	-	(Heterogeneous Workstations) ×16	-	-	[86]
KLT-JPEG2000	Transform-based (Lossy)	-	-	224	-	Nvidia GeForce GTX580 GPU	CUDA	-	[149]
KLT-JPEG2000	Transform-based (Lossy)	-	-	224	-	Nvidia GeForce GTX580 GPU	CUDA	-	[150]
CCSDS 123	Prediction-based (Lossless)	-	179.7	3.04	59.1	Xilinx V-5QV FX130T FPGA	VHDL	BIP	[82]
CCSDS 123	Prediction-based (Lossless)	-	116.0	0.95	122.1	Xilinx V-4 XC2VFX60 FPGA	VHDL	BIP	[82]
CCSDS 123	Prediction-based (Lossless)	-	219.4	5.30	41.3	Xilinx V-7 XC7VX690T FPGA	VHDL	BIP	[82]
CCSDS 123	Prediction-based (Lossless)	-	62.2	65	0.96	Nvidia GT 440 GPU	OpenCL	BIP	[82]
CCSDS 123	Prediction-based (Lossless)	-	62.6	29	2.2	Nvidia GT 610 GPU	OpenCL	BIP	[82]
CCSDS 123	Prediction-based (Lossless)	2.5	23.3	0.55	42.4	Xilinx Virtex-4 FPGA	VHDL	All	[96]
CCSDS 123	Prediction-based (Lossless)	2.5	47.6	-	-	Xilinx Virtex-7 FPGA	VHDL	All	[96]

**Table 14:** Comparison of the proposed compression system validated using the AVIRIS Indian Pines image.

Compression Algorithm	Algorithm Class	CR	Throughput (MSps)	Power (Watts)	Efficiency (MSps/W)	Hardware Platform	Programming Method	Scanning Order	Ref.
LCE	Prediction-based (Lossy)	-	12	225	0.05	Nvidia Tesla C2075 GPU	CUDA	BSQ	[116]
LCE	Prediction-based (Lossy)	-	130	225	0.6	Nvidia Tesla C2075-GeForce GTX 480 GPUs	CUDA	-	[55]
LCE	Prediction-based (Lossy)	-	130	250	0.52	Nvidia GeForce GTX 480 GPU	CUDA	-	[55]
Adaptive predictive-LCE	Prediction-based (Lossy)	2.7–3.2–1.6	120–100–110	225	0.53–0.44–0.5	Nvidia Tesla C2075 GPU	CUDA	BSQ	[117]
CCSDS 123	Prediction-based (Lossless)	2.3	3.5	0.169	20.7	Microsemi RTAX FPGA	VHDL	BSQ	[80]
CCSDS 123	Prediction-based (Lossless)	2.3	11.3	2.345	4.8	Xilinx Virtex-4 FPGA	VHDL	BSQ	[80]
CCSDS 123	Prediction-based (Lossless)	2.3	11.2	2.345	4.8	Xilinx Virtex-5 FPGA	VHDL	BSQ	[80]

**Table 15:** Comparison of the proposed compression system validated using the AVIRIS Lunar Lake image.

Compression Algorithm Details	Algorithm Class	CR	Throughput (MSps)	Power (Watts)	Efficiency (MSps/W)	Hardware Platform	Programming Method	Scanning Order	Ref.
JPEG-LS	Transform-based (Lossless)	2	-	250	-	Nvidia GTX480 GPU	CUDA	-	[143]
ANN-NLPCA	Learning-based (Lossy)	-	-	-	-	Intel Sandy Bridge GPU	-	-	[39]
Graph theory–Online-learning dictionary	Learning-based (Lossy)	5.3	-	-	-	Nvidia GPU	-	-	[48]

**Table 16:** Comparison of the proposed compression system validated using the Hyperion imager.

Compression Algorithm	Algorithm Class	CR	Throughput (MSps)	Power (Watts)	Efficiency (MSps/W)	Hardware Platform	Programming Method	Scanning Order	Ref.
Anomaly detection-wavelet-based transform	Transform-based (Lossy)	100	-	-	-	DSP-Xilinx XCV1000-XCV300 FPGAs	-	-	[40]
Anomaly detection-JPEG2000	Transform-based (Lossy)	25	-	-	-	DSP-Xilinx XCV1000-XCV300 FPGAs	-	-	[141]
CCSDS 123	Prediction-based (Lossless)	-	147	0.295	498.3	Xilinx Zynq-7020 SoC	VHDL	BIP	[134]
Autoencoder	Learning-based (Lossy)	-	-	-	-	JetStream Cloud Services	Openstack	-	[46]
CCSDS 123	Prediction-based (Lossless)	-	750	0.515	1456	Xilinx Zynq-7035 SoC	VHDL	BIP	[79]

**Table 17:** Ranking of the best six hardware-accelerated compression algorithms for HSI in terms of their efficiency.

Rank	Efficiency (MSps/W)	Throughput (MSps)	Power (Watts)	CR	Compression Algorithm Details	Algorithm Class (Compression Type)	Hardware Platform	Programming Method	Scanning Order	Ref. (Year)
1	**1456**	**750**	0.515	-	CCSDS 123	Prediction-based (Lossless)	Xilinx Zynq-7035 SoC	VHDL	BIP	[79] (2019)
2	498.3	147	**0.295**	-	CCSDS 123	Prediction-based (Lossless)	Xilinx Zynq-7020 SoC	VHDL	BIP	[134] (2018)
3	366.5	210	0.573	2.8	VS-3DGAP-ExtRice (CCSDS based)	Prediction-based (Lossless)	Xilinx Virtex-5 FPGA	Matlab-AccelDSP	-	[93] (2009)
4	231.4	162	0.7	4	LCPLC	Prediction-based (Lossy)	Xilinx Virtex-7 FPGA	VHDL and Verilog	BSQ	[60] (2020)
5	150.4	391	2.6	**8**	HYCA	CS-based (Lossy)	Xilinx Zynq-7020 SoC	VHDL	BIL	[151] (2020)
6	122.1	116.0	0.95		CCSDS 123	Prediction-based (Lossless)	Xilinx Virtex-4 FPGA FPGA	VHDL	BIP	[82] (2017)

The boldface indicates the best obtained value for each metric among the six studies.

## Data Availability

Data sharing is not applicable to this review.
